# *Rickettsia parkeri* hijacks tick hemocytes to manipulate cellular and humoral transcriptional responses

**DOI:** 10.3389/fimmu.2023.1094326

**Published:** 2023-02-10

**Authors:** Abdulsalam Adegoke, Jose M. C. Ribeiro, Sidney Brown, Ryan C. Smith, Shahid Karim

**Affiliations:** ^1^ School of Biological, Environmental, and Earth Sciences, The University of Southern Mississippi, Hattiesburg, MS, United States; ^2^ Vector Biology Section, Laboratory of Malaria and Vector Research, National Institute of Allergy and Infectious Diseases, National Institutes of Health, Rockville, MD, United States; ^3^ Department of Plant Pathology, Entomology, and Microbiology, Iowa State University, Ames, IA, United States

**Keywords:** Hemocytes, clodronate liposome, phagocyte, *Rickettsia parkeri*, transcriptome, *nimrod B2*, *eater*, *Amblyomma maculatum*

## Abstract

**Introduction:**

Blood-feeding arthropods rely on robust cellular and humoral immunity to control pathogen invasion and replication. Tick hemocytes produce factors that can facilitate or suppress microbial infection and pathogenesis. Despite the importance of hemocytes in regulating microbial infection, understanding of their basic biology and molecular mechanisms remains limited.

**Methods:**

Here we combined histomorphology and functional analysis to identify five distinct phagocytic and non-phagocytic hemocyte populations circulating within the Gulf Coast tick *Amblyomma maculatum*.

**Results and discussion:**

Depletion of phagocytic hemocytes using clodronate liposomes revealed their function in eliminating bacterial infection. We provide the first direct evidence that an intracellular tick-borne pathogen, *Rickettsia parkeri*, infects phagocytic hemocytes in *Am. maculatum* to modify tick cellular immune responses. A hemocyte-specific RNA-seq dataset generated from hemocytes isolated from uninfected and *R. parkeri*-infected partially blood-fed ticks generated ~40,000 differentially regulated transcripts, >11,000 of which were immune genes. Silencing two differentially regulated phagocytic immune marker genes (*nimrod B2* and *eater*-two *Drosophila* homologs), significantly reduced hemocyte phagocytosis.

**Conclusion:**

Together, these findings represent a significant step forward in understanding how hemocytes regulate microbial homeostasis and vector competence.

## 1 Introduction

Ticks are major vectors for bacterial, viral, and protozoan pathogens of human and veterinary importance. The Gulf Coast tick *Amblyomma* (*Am.*) *maculatum* is a competent vector of the spotted fever group *Rickettsia parkeri*, an obligate intracellular bacterium that causes an eschar-like lesions at the site of tick attachment (1, 2). Vector competence is influenced by the ability of a pathogen to establish infection, replicate, and disseminate across vector tissues, processes counteracted by the arthropod’s innate immune system. Similar to vertebrates, the tick immune system has both cellular and humoral arms. The cellular arm is represented by hemocytes, which are professional immune cells equivalent to vertebrate leukocytes. Following microbial infection, tick hemocytes execute cell-mediated responses including phagocytosis, encapsulation, and nodulation, clearing microbes from the system (3–6). The humoral arm of the tick immune system produces soluble effector molecules that activate the complement pathway, prophenoloxidase pathway, and melanization cascade and produce reactive oxygen and nitrogen species (7–11). Hemocytes also secrete effectors that eventually activate humoral responses (9, 12).

Tick hemocytes have historically been classified into prohemocytes, plasmatocytes, granulocytes, spherulocytes, and oenocytoids (13) based on their quantity, size, shape, nuclear-cytoplasmic ratio, and presence of inclusion bodies. Recent ultrastructural studies have narrowed the classification to prohemocytes, granulocytes I, and granulocytes II (14, 15), and functional characterization of tick hemocytes established the existence of core invertebrate hemocyte functions. Both hard and soft tick hemocytes use phagocytosis as the primary defense mechanism (3, 16–18), especially in the granulocyte and plasmatocyte subsets (8, 16, 19, 20). Hemocytes can phagocytose tick-transmitted pathogens, such as hemocyte engulfment of *Borrelia* spirochetes, in a process described as coiling phagocytosis (21). *Ixodes scapularis* hemocytes were also reported to be infected with *Anaplasma phagocytophilum*, a requirement for subsequent salivary gland infection (22). The release of effector molecules complements hemocyte-mediated responses as part of the humoral defense response and include several pathogen recognition molecules such as lectins, antimicrobial peptides (AMPs), and thioester-containing proteins (23–25). However, the molecular mechanisms underlying tick hemocyte-pathogen interactions remain largely uncharacterized.

Here we used a conservative immunofluorescence and morphological approach to classify hemocyte subpopulations in *Am. maculatum*. Through *in vivo* phagocytosis of fluorescent beads, we functionally differentiate phagocytic from non-phagocytic hemocytes. For the first time, we demonstrate depletion of phagocytic hemocyte subsets using clodronate liposomes (CLD) in a tick species, thereby defining a role for phagocytic hemocytes in innate immune responses against bacterial challenge. Next-generation transcriptome analysis of uninfected and *R. parkeri-*infected hemolymph samples reveals molecular changes associated with pathogen recognition, immune pathway activation, and hemocyte production, amongst others. Using RNA interference, we characterize two previously unreported molecular markers of hemocyte phagocytosis in ticks. We also show that *R. parkeri* infects phagocytic hemocytes, thereby possibly playing a vital role in the systemic dissemination of *R. parkeri* to other tissues.

## 2 Materials and methods

### 2.1 Tick maintenance and rearing

*Am. maculatum* ticks were maintained at the University of Southern Mississippi following previously established protocols for hard ticks (26). Established laboratory colonies of *Rickettsia parkeri-*infected ticks were generated from questing ticks. Unfed adult ticks were collected using the drag-cloth method during the summer months of 2019 from Mississippi Sandhill Crane, National Wildlife Refuge, Gautier, Mississippi (https://www.fws.gov/refuge/mississippi_sandhill_crane/). *R. parkeri-*free *Am. maculatum* ticks were obtained from the tick rearing facility at Texas A&M (TAMU, College Station, TX, USA) tick-rearing facility. These ticks were fed on cattle, which clears *R. parkeri* from tick tissues across feeding stages.

### 2.2 Hemolymph perfusion and hemocyte quantification

Before hemolymph collection, unfed or partially fed ticks were cleaned in a 10% sodium hypochlorite solution for 5 minutes, followed by a 10-minute wash in 70% ethanol and cleaning using double distilled water. Hemolymph was collected from ticks as previously described (27) using a freshly prepared modified citrate-EDTA anticoagulant buffer (vol/vol 60% Schneider’s *Drosophila* medium and 70% 5 mM EDTA in 1X PBS) (28) and kept on ice until needed. A modified perfusion method was used to collect hemolymph from unfed ticks. Briefly, ticks were placed with their ventral side facing downwards on double-sided tape mounted on a petri dish, which was then placed on ice for approximately 20 minutes to stimulate hemolymph flow within the tick. 0.5 µL of anticoagulant solution was injected into ticks between the basis capituli and scutum. Injected ticks were kept recovering at room temperature for 20 minutes before hemolymph perfusion. 1-2 incisions were made between the ridges of the festoon using a 33G removable needle (Hamilton Company, Franklin, MA, USA). Immediately, 5 µL of anticoagulant solution was injected from the basis capituli and the exiting hemolymph was collected in a 1.5 mL microcentrifuge tube. Perfused hemolymph was centrifuged at 500 rpm for 3 minutes at 4°C to pellet the hemocytes, and the supernatant was removed. Hemocyte pellets were resuspended in a fresh anticoagulant buffer and kept on ice until needed to allow for the collection of hemolymph from unfed ticks. Total hemocytes were quantified using the trypan blue exclusion method (Invitrogen, Thermo Fisher Scientific, Waltham, MA, USA). Briefly, perfused hemolymph was mixed with 0.4% trypan blue, and 10 μL of the mixture was pipetted into a cell counting chamber. Hemocytes were quantified using a Countess automated cell counter (Invitrogen, Thermo Fisher Scientific Waltham, MA, USA). The number of hemocytes was estimated by placing perfused hemolymph onto the grooves of an improved hemocytometer chamber (Bright-Line, Hausser, Scientific Horsham, PA, USA), and hemocytes were differentiated morphologically as described previously (29, 30). A total of five ticks was used for hemocyte quantification. Three separate count was carried out from individual ticks and the average represent the total hemocyte population from the tick.

### 2.3 Chemical depletion of phagocytic hemocytes

Phagocytic cells were depleted as previously described with slight modifications (31, 32). To deplete circulating phagocytic hemocytes from the tick’s hemolymph, clodronate liposomes (CLD) or control liposomes (LP) (Standard Macrophage Depletion Kit, Encapsula Nano Sciences LLC, Brentwood, TN, USA) were injected into the hemolymph using a 2.5 μL 600 series Hamilton microliter syringe connected to a 33G removable needle (Hamilton Company). The optimal concentration of CLD and LP necessary for depletion with no adverse effect on survival was initially determined by injecting CLD and LP (stock, 1:2, 1:5, and PBS) in 1X PBS into groups of 15 ticks. These ticks were then monitored for survival over eight days beginning 24 h post-injection. Subsequent depletion experiments were performed using 1:5 dilution of CLD and LP in 1X PBS.

### 2.4 Bacterial challenge and survival analysis

*Escherichia coli* (*E. coli*) strain DH5-alpha and *Staphylococcus aureus* (*S. aureus*) strain RN4220 were grown overnight at 37°C in LB and TSA media, respectively. Overnight cultures were carefully removed, centrifuged, and adjusted to a concentration of OD_600_ = 0.5 for *E. coli* and OD_600_ = 0.1 for *S. aureus* in 1X PBS. *R. parkeri* were cultured as previously described (33). Frozen stocks of *R. parkeri* were revived by infecting Vero cells. After the cells were revived and replicated, the concentration of *rickettsiae* was determined using the plaque assay. *Rickettsiae* were isolated from Vero cells by lysis using sonication (BioRuptor™ Pico, Denville, NJ, USA) for 5 minutes in cycles of 30 seconds on and 30 seconds off at 4°C. After sonication, the suspension was centrifuged at 1000 x g for 5 minutes at 4°C to pellet cell debris, and the supernatant was passed through a 0.22 µm syringe filter (Fisher Scientific, Grand Island, NY, USA). *R. parkeri* was stored in SPG medium on ice until ready for use. Ticks were challenged with 0.2 µL of 10^7^ *rickettsiae* 24 h post-CLD or LP injection. LPS and heat-killed *S. aureus* were used as positive controls for bacterial injection. The injection of sterile 1X PBS was used as a negative and injection control. Ticks were maintained and constantly monitored every 24 hours for signs of mortality.

### 2.5 *In vivo* phagocytosis assay

The phagocytic function of tick hemocytes was assessed by injecting fluorescent-conjugated carboxylated beads as previously reported (32, 34) with slight modifications to adapt for use in ticks. Briefly, ticks were injected with 2% (vol/vol) 0.2 µL of yellow-green Carboxylated-Modified Microspheres (Thermo Fisher Scientific, Waltham, MA, USA) diluted in anticoagulant buffer and allowed to recover in incubators for 4 hours at 22°C and 95% relative humidity (RH). Subsequently, hemolymph was perfused using an anticoagulant solution (vol/vol 60% Schneider’s *Drosophila* medium and 70% 5 mM EDTA in 1X PBS). Perfused hemolymph was allowed to adhere on a glass microscope slide for 1 h at room temperature. Hemocytes were fixed in 4% paraformaldehyde (PFA) in 1X PBS for an additional hour. Fixed hemocytes were washed with 1X PBS. Hemocytes were incubated with 20 μM Hoechst 33342 (Thermo Fisher Scientific) diluted in 1X PBS and 75 μM Vybrant CM-Dil (Invitrogen, Carlsbad, CA, USA) for 1 hour at RT, after which slides were washed three times in 1X PBS and allowed to dry. Slides were mounted on a coverslip in 10 μL Fluoromount-G mounting medium (SouthernBiotech, Birmingham, AL, USA). Vybrant CM-Dil is a dye that stains the hemocyte cell membrane thus providing conclusive evidence for the presence of the injected beads within or outside of the hemocytes. A total of five ticks were used to quantify phagocytic hemocytes. For each tick, three separate fields were counted (200 hemocytes per field of view) and the proportion of phagocytic hemocytes out of the total number of hemocytes was estimated.

### 2.6 In-vivo EdU incorporation assay

We estimated hemocyte differentiation by visualizing and quantifying the synthesis of new DNA *in vivo* based on 5-ethynyl-2′-deoxyuridine (EdU) incorporation and subsequent detection using the Click-iT EdU Alexa Fluor 647 kit (Invitrogen, Grand Island, NY, USA) as previously described (35, 36), with the only exception that 0.5 µL of 20 mmol l−1 EdU in anticoagulant buffer was injected into ticks. Ticks were allowed to recover in incubators for 4 hour at 22°C and 95% RH. Following recovery, hemolymph was perfused from ticks and allowed to attach on a microscope glass slide for 1 hour at 4°C. Hemocytes were subsequently fixed with 4% paraformaldehyde diluted in 1X PBS for 30 minutes at RT, washed three times with 3% bovine serum albumin (BSA) in 1X PBS, permeabilized for 30 min with 0.5% Triton-X in PBS at RT, followed by another wash step with 3% BSA in 1X PBS. Hemocyte slides were subsequently incubated in the dark with the Click-iT reaction cocktail for 30 minutes at RT according to the manufacturer’s instructions, followed by a wash step with 3% BSA in 1X PBS. Hemocyte slides were subsequently incubated with 20 μM Hoechst 33342 (Thermo Fisher Scientific) diluted in 1X PBS for 1 hour at RT, after which slides were washed three times in 1X PBS and allowed to dry before mounting on a microscope glass slide by adding 10 μL Fluoromount-G mounting medium (SouthernBiotech).

### 2.7 Hemocyte staining

Tick hemolymph was perfused into an anticoagulant buffer and allowed to adhere to a glass coverslip in a humid chamber at 4°C for 1 hour. Without washing, hemocytes were fixed by adding 4% PFA solution in 1X PBS for an additional 1 hour at RT. After fixation, cells were washed three times with 1X PBS and permeabilized with 0.1% Triton X-100 for 1 hour at RT. Without washing, hemocytes were blocked in 1% BSA solution in 0.1% Triton X-100 for an additional 1 hour at RT. Excess blocking solution was washed with 1X PBS. Hemocytes were incubated with 1U phalloidin (Alexa Fluor™ 488 Phalloidin, Molecular Probes, Thermo Fisher Scientific) and 20 μM Hoechst 33342 (Molecular Probes, Thermo Fisher Scientific) diluted in 1X PBS for 1 hour at RT, after which slides were washed in 1X PBS and allowed to dry before mounting on a microscope glass slide by adding 10 μL Fluoromount-G mounting medium (SouthernBiotech).

### 2.8 RNA extraction, cDNA synthesis, and qRT-PCR

Hemolymph was collected from 10 individual ticks as described above and pooled, and an anticoagulant buffer was added to a total volume of 250 μL. RNA was extracted using the Trizol-chloroform separation and isopropanol precipitation method with slight modifications. Following the initial chloroform separation of RNA into the aqueous phase, a second separation was performed by adding a 1:1 volume of chloroform to the aqueous phase, centrifuging at maximum speed (19,980 RCF), and the transparent upper phase was used to proceed with isopropanol precipitation. Second, an ethanol wash of the RNA pellet was carried out twice to help completely remove the isopropanol carryover. The RNA pellet was air dried and resuspended in 30 μL of nuclease-free water, concentration and quality checked, and stored at -80°C until use. Complementary DNA synthesis and qRT-PCR were conducted as previously described (37). Sequences of gene-specific primers designed to amplify cDNA fragments are listed in **Supplementary Table S1**. Transcriptional gene expression was normalized against the *Am. maculatum β-actin* gene. The synthesized cDNA was used to measure mRNA levels by qRT-PCR using the CFX96 PCR Detection System (Bio-Rad Inc., Hercules, CA, USA) described previously (37–39).

### 2.9 Double-stranded RNA synthesis and delivery

Double-stranded RNA from the homologs of *Drosophila nimrod B2* and *eater* transcripts was synthesized for gene silencing and microinjected into unfed adult female *Am. maculatum* ticks as previously described (37–41). Before injection, dsRNA targeting each gene was diluted to a working concentration of 1 μg/μL in nuclease-free water. Double-stranded RNA from the green fluorescent protein (*Gfp*) gene was synthesized and injected as an irrelevant control.

### 2.10 Illumina sequencing

RNA samples from uninfected and *R. parkeri-*infected *Am. maculatum* hemolymph were sent for sequencing by Novogene (China). Briefly, partially blood-fed (~50 mg, slow blood feeding phase and ~200 mg, start of fast feeding phase) ticks were selected for RNA sequencing. Hemolymph was collected from 120 partially blood-fed ticks during the slow-feeding (~50 mg) and fast-feeding phases (~200 mg). Three biological replicates of hemolymph from the slow-feeding and fast-feeding phases were included in each sample of the *R. parkeri-*infected or uninfected group, i.e., a total of 12 samples. Hemolymph from ten partially-fed ticks was combined for each biological replicate. Hemolymph RNA was extracted as described above. RNA libraries were constructed from hemolymph RNA from six uninfected and six *R. parkeri-*infected ticks using the NEBNext Ultra™ RNA library Prep Kit (New England Biolabs, Ipswich, MA, USA). RNA library preparation and sequencing were conducted by Novogene Co., Ltd. (Beijing, China).

### 2.11 Bioinformatics analysis

Raw reads were stripped of contaminating primers, and bases with qual values <20 were trimmed. Clean reads were assembled using the Abyss (42) and Trinity (43) assemblers. Resulting contigs were re-assembled with a blastn and cap3 assembler (44) pipeline as described previously (45). Coding sequences were extracted based on blastx results derived from several database matches, including a subset of the non-redundant NCBI protein database containing tick and other invertebrate sequences, as well as the Swiss-Prot and Gene Ontology (GO) databases. All open reading frames larger than 200 nucleotides were extracted, and those matching known proteins or with a signal peptide were retained. The resulting peptide and coding sequences were mapped to a hyperlinked spreadsheet including blastp and rpsblast matches to several databases and an indication of the signal peptide (46), transmembrane domains (47), and O-galactosylation sites (48). edgeR was used in ancova mode to detect statistically significant differentially-expressed genes according to feeding or infection status (49). edgeR inputted the read matrix for genes with at least one library expressing an FPKM (fragments per thousand nucleotides per million reads) equal to or larger than 10. For heat map visualization of CDS temporal expression, Z scores of the FPKM values were used. All deduced coding sequences and their reads are available for browsing with hyperlinks to several databases (**Supplementary Table S2**).

### 2.12 RNA-seq and differential gene expression analysis

As previously described, differentially expressed genes from edgeR analysis were analyzed using the iDEP (integrated Differential Expression and Pathway analysis) online tools (50). The expression matrix representing read counts of differentially-expressed genes and the gene IDs were uploaded to the iDEP user interface and used for data exploration.

### 2.13 Immunofluorescence of R. parkeri

Hemolymph from unfed or partially fed ticks was perfused onto a microscope coverslip, and hemocytes were allowed to adhere for 1 hour at RT. Hemocytes were fixed in 4% PFA (4% in PBS; J19943-K2, Thermo Fisher Scientific) for 30 minutes at RT. Coverslips containing hemocytes were permeabilized with 0.1% Triton X-100 for 30 minutes at RT. For non-permeabilized coverslips, 1X PBS was added to the hemocytes for 30 minutes at RT. This step was followed by three times washing with 1X PBS. Non-specific proteins were blocked with 1% BSA solution in PBS for 1 hour, followed by primary incubation with mouse anti-*Rickettsia* M14-13 (generously provided by T. Hackstadt, NIH/NIAID Rocky Mountain Laboratories (51, 52), and rabbit anti-*Sca2* (generous gift from Matthew D. Welch, UC Berkeley). A no primary antibody control sample (negative control) was prepared in parallel. *R. parkeri* was detected using goat anti-rabbit Alexa Fluor 568 and goat anti-mouse Alexa Fluor 568 (1:500 in 1% BSA; Invitrogen, Thermo Fisher Scientific). Samples were washed three times in 1X PBS to remove free antibodies. Hemocytes were incubated with 20 μM Hoechst 33342 (Molecular Probes, Thermo Fisher Scientific) diluted in 1X PBS for 1 hour at RT, after which slides were washed three times in 1X PBS and allowed to dry before mounting on a microscope glass slide by adding 10 μL Fluoromount-G mounting medium (SouthernBiotech). To test for lysosomal activity, hemocytes from *R. parkeri* infected ticks were stained with the acidotropic dye LysoTracker Red (Invitrogen) for 1 hour at room temperature, washed thrice in PBS and fixed with 4% paraformaldehyde for 30 minutes. Hemocytes were then processed for *R. parkeri* immunostaining as described previously

### 2.14 Imaging acquisition

Confocal images were acquired with a Leica STELLARIS STED (Leica Microsystems, Wetzlar, Germany) confocal microscope using either a 40X, 63X, or 100X objective (zoom factor 3-5; numerical aperture of 1). Images were obtained using both sequential acquisition and variable z-stacks. The 405 UV laser was used to acquire the DAPI channel, while the tunable white light laser (WLL) was used to capture the Alexa-Fluor channel. A z-stack of the images consisting of 150-250 slices was compiled for all images captured, and the proprietary Leica built-in post-processing plugin was used for deconvolution and to carry out lightning processing. All images were exported as acquired and compiled in PowerPoint software.

## 3 Results

### 3.1 Discrimination of hemocyte types

Light microscopic examination of direct hemolymph smears revealed a heterogeneous hemocyte population. There were two distinct small and large cell populations, the latter comprising cells with varying cytoplasmic contents, nuclear shape, and cytoplasm size (**Supplementary Figures S1A, B**). The position of the nucleus was variable: certain hemocytes possessed large, centrally placed nuclei occupying most of the cytoplasmic space, while in some cells the nucleus was peripheral and binucleated. Variable granulation was also observed in the cytoplasm of some hemocyte types.

**Figure 1:**
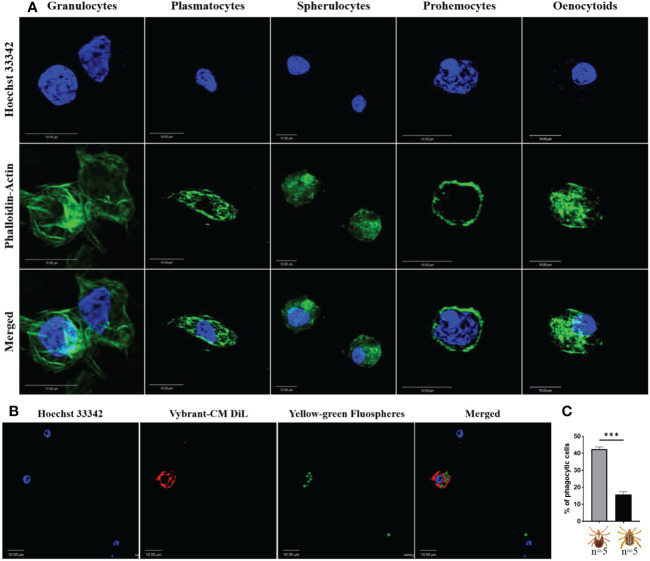
Confocal microscopy images of *Am. maculatum* hemocytes stained with phalloidin (green) and Hoechst 33342 (blue). Hemocytes were subtyped based on nuclear size and location and cytoplasmic projections **(A)**. Granulocytes are relatively large and have multiple actin projections. Plasmatocytes are pyriform and have a centrally placed nucleus. Spherulocytes possess a peripherally placed nucleus. Prohemocytes are characterized by a relatively large nuclear to cytoplasmic ratio and oenocytoids by a smaller nuclear to cytoplasmic ratio. Hemocytes were incubated with Hoechst 33342 (blue) and Alexa Fluor™ 488 Phalloidin (green) that labels the nucleus and actin respectively. Representation of a phagocytic hemocyte following injection of green FluoSpheres and subsequent staining of the nucleus with Hoechst 33342 (blue) and membrane with Vybrant-CM Dil (Red) **(B)** and the quantification of phagocytic hemocytes in male and female ticks **(C)**. Quantitative data were analyzed using unpaired *t-*tests in GraphPad Prism v8.4.1. ***P < 0.001. Scale bar = 10 μm.

However, the resolution of light microscopy limited our discrimination of hemocyte subsets. We therefore assessed whether we could further classify hemocytes using commonly used fluorescent markers. Perfused hemolymph was stained with wheat germ agglutinin (WGA), Vybrant CM-Dil (a lipophilic cell membrane stain), and Hoechst 33342 (a nuclear stain). WGA discriminated hemocyte populations with varying degrees of binding intensity, indicating a potential difference in hemocyte function based on their lectin binding activity. By contrast, all hemocytes were positive for CM-Dil (**Supplementary Figure S1C**). To further differentiate between hemocyte subtypes, we co-stained with phalloidin (an actin stain) and DAPI (a nuclear stain). Five distinct hemocyte types were identified based on shape, actin projections, and nuclear-cytoplasmic ratio: (i) granulocytes were relatively large and had multiple actin projections; (ii) plasmatocytes were pyriform with a centrally placed nucleus; (iii) spherulocytes possessed a peripherally placed nucleus; (iv) prohemocytes were characterized by a relatively high nuclear to cytoplasmic ratio; and (v) oenocytoids had a smaller nuclear to cytoplasmic ratio (**Figure 1A**).

The total hemocyte population differed significantly between partially fed and unfed female ticks, with hemocyte numbers increasing after the blood meal (**Supplementary Figure S1D**). Feeding also affected the hemocyte subtype distribution in male and female ticks: feeding significantly increased granulocyte numbers in females (**Supplementary Figure S1E**) and significantly decreased the spherulocyte population in both male and female ticks (**Supplementary Figure S1F**). Plasmatocyte numbers were lower in blood-fed females than unfed females but increased in males on feeding (**Supplementary Figure S1G**). There was a trend to oenocytoids numbers increasing following feeding in both males and females (**Supplementary Figure S1G**), but prohemocytes were absent in both male and female ticks following feeding (**Supplementary Figure S1I**).

Hemocyte populations were histomorphologically similar in male and female ticks, so we next examined functional differences between male and female hemocytes by assessing their phagocytic abilities. The proportion of phagocytic hemocytes were higher in female ticks than in male ticks in an *in vivo* phagocytosis assay of yellow-green FluoSpheres (**Figures 1B, C**). Taken together, these data demonstrate that *Am. maculatum* hemocyte heterogeneity might influence hemocyte function.

### 3.2 Clodronate liposomes deplete and impair phagocytic hemocyte functions

**Figure 2:**
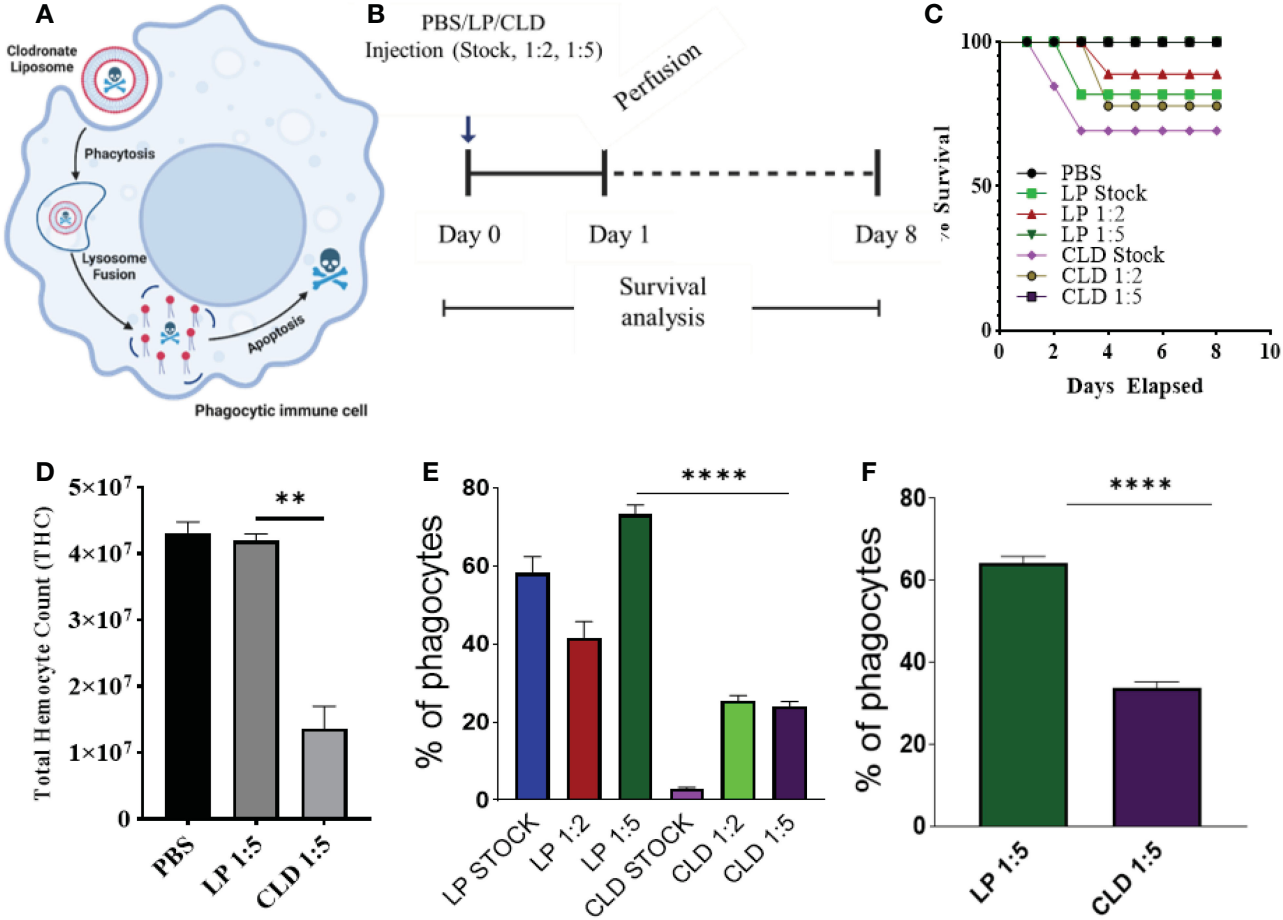
Clodronate depletion of phagocytic tick hemocytes and validation of phagocyte depletion. Mechanism of clodronate liposome-induced depletion of professional phagocytes **(A)**. Schematic showing the optimization of clodronate and liposome concentrations to deplete phagocytic hemocytes **(B)**. Tick survival was evaluated following injection of clodronate (CLD) and control liposomes (LP) at different concentrations (stock, 1:2, 1:5 in 1X PBS), with 1X PBS used as control **(C)**. Hemolymph was perfused 24 h post-CLD or LP injection (unfed status) to assess the effect of depletion on total hemocyte count **(D)** and proportion of phagocytic hemocytes **(E)**. The proportion of phagocytic hemocytes was also assessed in CLD- or LP-injected ticks 5-days post feeding **(F)**. Survival was checked each day for 8 days; 15 ticks were assigned to each treatment group. Significance was determined with the log-rank (Mantel-Cox) test using GraphPad Prism v8.4.1. Error bars represent ± SEM of five ticks. Ticks from PBS, LP 1:5 and CLD 1:5 injected groups all survived throughout the observation period. Quantitative data were analyzed using unpaired *t-*tests in GraphPad Prism v8.4.1. **P < 0.01, ****P < 0.0001.

In the absence of definitive molecular markers of hemocyte subtypes in ticks, there is a need for alternative tools to study the role of hemocytes in cellular immunity and vector competence. To this end, clodronate liposomes (CLD), a pharmacological agent that specifically targets and depletes professional phagocytes *via* apoptosis (31, 32, 53, 54), was used to deplete phagocytic hemocytes in tick hemolymph. CLD exclusively targets cells with phagocytic abilities (55). Upon phagocytosis, the liposome is degraded by the lysosome, which releases toxic clodronate to promote apoptosis (**Figure 2A**).

We first tested different clodronate and control liposome (LP) concentrations to determine their impact on tick survival (**Figure 2B**). Injection of CLD or LP at a 1:5 dilution had no adverse impact on tick survival (**Figure 2C**) but significantly reduced total hemocyte populations (**Figure 2D**) due to reduced numbers of phagocytic hemocytes (**Figure 2E**). To determine the effect of the blood meal on phagocytic hemocyte depletion, we injected unfed ticks with a 1:5 dilution of CLD or LP before a blood meal and quantified the phagocytic hemocyte population following partial feeding. The blood meal did not interfere with the ability of CLD to deplete phagocytic hemocytes (**Figure 2F**).

**Figure 3:**
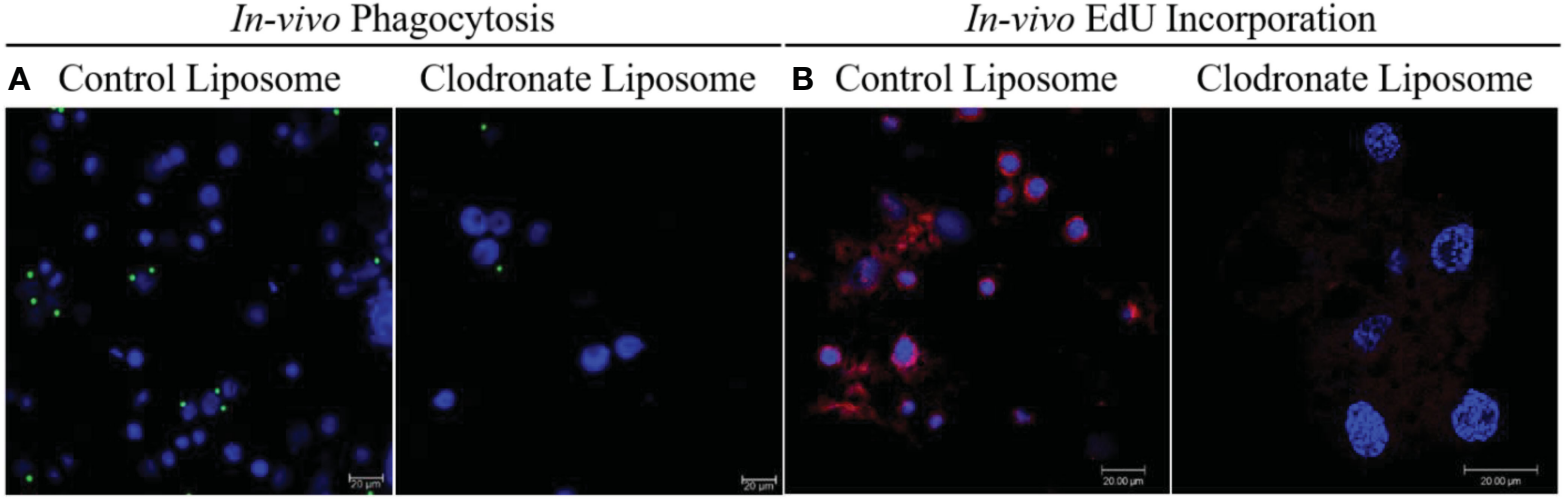
Clodronate liposomes deplete hemocyte functions. Clodronate liposomes impaired hemocyte phagocytosis **(A)** and interfered with EdU incorporation into hemocytes DNA **(B)**. Scale bar = 20 μm.

To further assess the effect of CLD depletion on phagocyte function, ticks injected with CLD or LP were fed and *in vivo* hemocyte phagocytosis assayed with yellow-green FluoSpheres. Hemolymph of LP-injected ticks contained more hemocytes that engulfed one or two FluoSpheres than hemocytes from clodronate-depleted ticks (**Figure 3A**). Hemocyte populations have previously been shown to increase following a blood meal due to cellular division (36), so we determined the impact of clodronate depletion on hemocyte DNA replication. Partially blood-fed CLD or LP-injected ticks were injected with EdU, and their hemocytes were assayed for EdU incorporation into hemocyte DNA. Significantly more EdU was incorporated into LP-injected tick hemocytes compared with CLD-injected ticks (**Figure 3B**). Together, these data confirm effective chemical depletion of the phagocytic hemocyte population and show that CLD interferes with the abilities of phagocytic hemocytes to undergo replication.

### 3.3 Depletion of phagocytic hemocytes impairs survival against bacterial challenge

**Figure 4:**
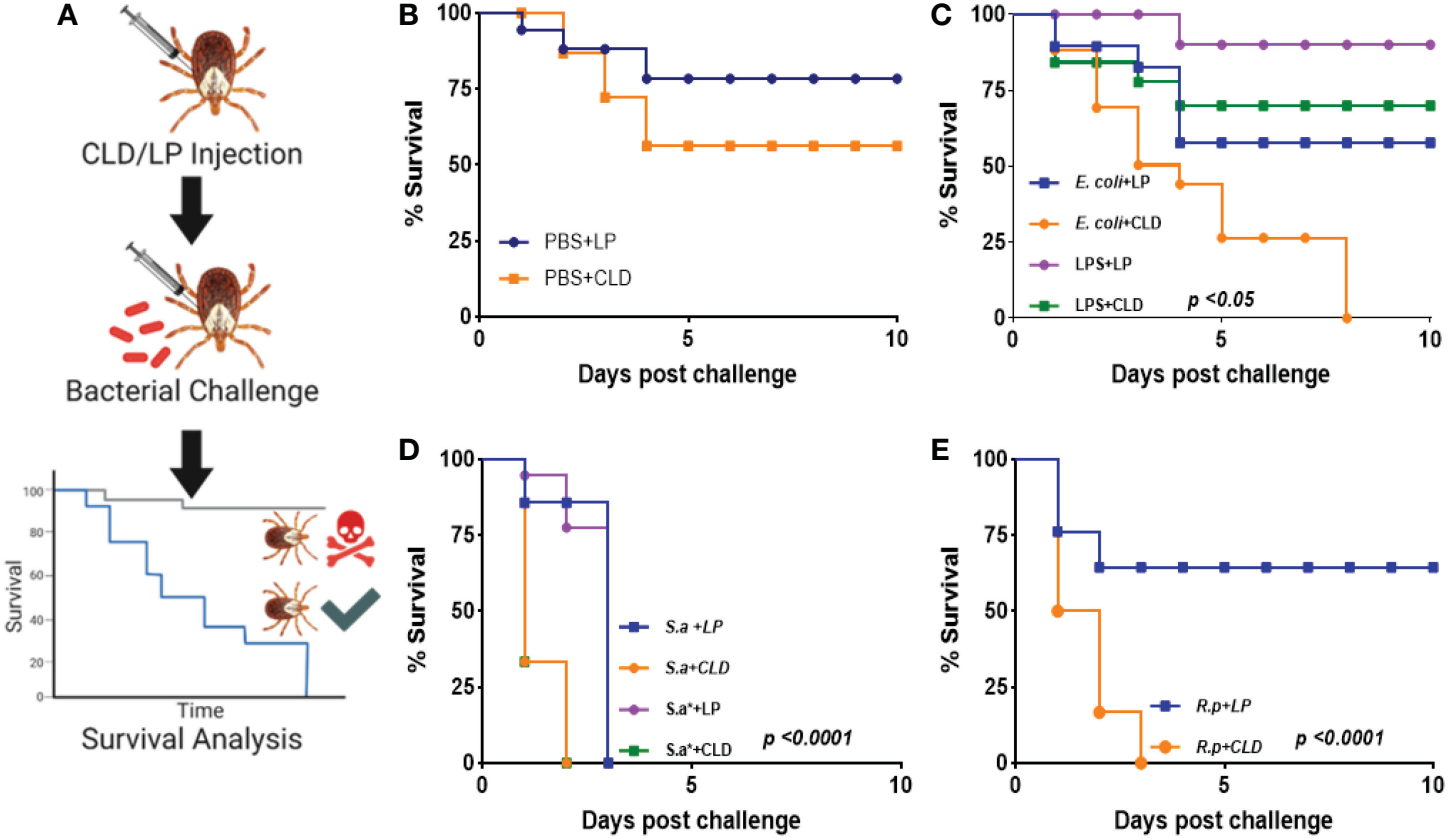
Depletion of phagocytic hemocytes impairs survival against bacterial challenges. Unfed female *Am. maculatum* were injected with either LP or CLD at 1:5 dilution and 24 h later challenged with bacteria or sterilely injured **(A)**. Tick survival was monitored every 24 h for 10 days to evaluate the effect of sterile injury **(B)**, *E*. *coli* **(C)**, *S. aureus* **(D)**, or *R. parkeri* **(E)** challenge. Data were analyzed with the log-rank (Mantel-Cox) in GraphPad Prism v8.4.1. S.a: live S. aureus, S.a*: heat-killed S. aureus, R.p: R. parkeri.

Hemocytes are a vital defense mechanism against invading microbes in ticks, mosquitoes, and *Drosophila* (32, 33). We therefore attempted to determine how immunocompromised ticks would survive challenge with both Gram-positive and Gram-negative bacteria (**Figure 4A**). Ticks were unaffected by injection with PBS (**Figure 4B**). Phagocyte depletion impaired tick survival against Gram-negative *E. coli* (**Figure 4C**), but Gram-positive *S. aureus* (live and heat-inactivated) significantly affected tick survival in both CLD and LP-treated groups (**Figure 4D**). Similarly, phagocyte depletion significantly impaired survival against *Am. maculatum-*transmitted *R. parkeri* (**Figure 4E**). Since phagocytic granulocytes act as scavengers of invading microbes (6, 31, 32), these data further support the hypothesis that phagocytic hemocytes are critical components of the immune response in ticks and maintain tick microbial homeostasis *via* cell-mediated immunity and interactions with pathogenic microbes (6, 56).

### 3.4 *R. parkeri* can infect circulating hemocytes

**Figure 5:**
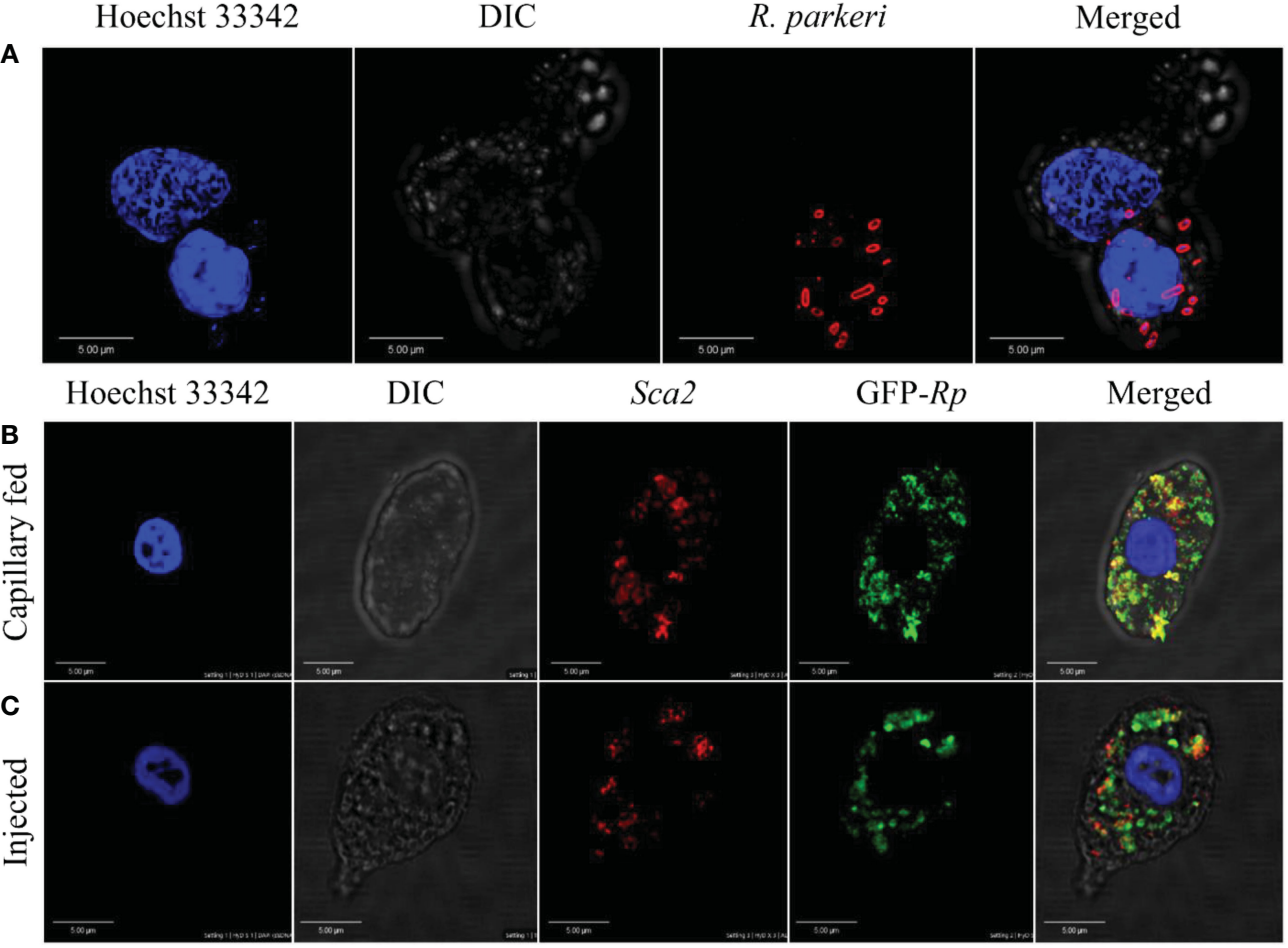
Confocal microscopy images of phagocytic hemocytes infected with *Rickettsia parkeri*. Representative confocal images of immunofluorescence staining for *R. parkeri* proteins showing hemocytes from natural and artificially infected ticks. **(A)** Immunolocalization of *R. parkeri* in hemocytes of naturally infected *Am. maculatum*. Hemocytes were incubated with primary antibodies targeting *R. parkeri* outer membrane protein (red) and Hoechst 33342 (blue). Infection of hemocytes with *R. parkeri* following **(B)** capillary feeding and **(C)** microinjection of GFP-expressing *R. parkeri* into uninfected *Am. maculatum*. Hemocytes were incubated with *R. parkeri* Sca2 antibody (red) and Hoechst 33342 (blue). Hemocytes were fixed, permeabilized and blocked prior to incubating with antibody. Scale bar = 5 μm.

The tick hemolymph contains a heterogeneous population of circulating hemocytes, as shown by ourselves (**Figure 1A**) and others (4, 7, 10, 15, 30, 57–59). *R. parkeri* acquired during a blood meal must circumvent both cellular and tissue barriers in the midgut to access hemolymph for systemic dissemination and subsequent transmission to a mammalian host. In the hemolymph, *R. parkeri* must either avoid, evade, or suppress hemocyte-mediated immune responses to successfully disseminate. *R. parkeri* is closely related to *Anaplasma* (*A.*) *phagocytophilum*, both existing as obligate intracellular pathogens and belonging to the same Rickettsiales order. Dissemination of *A. phagocytophilum* to the salivary gland in its tick vector is facilitated by direct hemocyte infection following midgut colonization by the bacteria (22). Since our understanding of how *R. parkeri* disseminates through the hemolymph from the midgut to other tissues is still limited, we asked whether *R. parkeri* can infect circulating hemocytes. Hemolymph from infected and unfed female *Am. maculatum* were incubated with primary antibodies targeting the outer membrane of *R. parkeri*, and *R. parkeri* was detected in the cytoplasm of hemocytes (**Figure 5A**). In addition, intracytoplasmic infection was detected in the hemocytes of uninfected ticks previously injected or capillary-fed with GFP-expressing *R. parkeri* (**Figures 5B, C**). While positive Sca2 staining suggested the presence of *R. parkeri* in hemocytes, this could also have occurred through binding of *R. parkeri* to the surface of hemocytes arising from the immune response. However, detection of *R. parkeri* Sca2 signal in both permeabilized and unpermeabilized hemocytes further confirmed active entry into hemocytes. Lysosomal imaging of *R. parkeri* infected hemocytes showed very few bacteria associated with the lysosomal compartments (**Supplementary Figure S3**). Intracellular, pathogenic bacteria avoid several host vacuoles to access and replicate in the host cytosol and spread from cell to cell. Internalized bacteria inside the host cells are ingested into lysosomal compartments where the highly acidic compartment degrades them. A recent study showed *R. parkeri* utilizes a Patatin-like phospholipase to avoid cytoplasmic vacuoles and evade autophagy in the mammalian host (60). These data argue that tick hemocytes - and potentially phagocytic hemocytes - are infected by *R. parkeri*, which might be important for its systemic dissemination. Similar findings had been reported with *A. phagocytophilum* and Zika virus infection of tick and mosquito hemocytes, respectively (61), further corroborating our observations.

### 3.5 RNA-seq reveals changes in hemocyte gene expression associated with R. parkeri infection

Identifying hemocyte-specific transcripts is crucial for the discovery of immune factors participating in hemocyte-mediated immune responses. While tick hemocyte transcripts have previously been characterized (62), the effect of pathogen infection on tick hemocyte gene expression is unknown. Here we generated and compared hemocyte transcripts from *R. parkeri*-infected (n = 6) and uninfected (n = 6) *Am. maculatum.* Assembly of the 305,276,990.3 reads from 12 libraries allowed us to identify 37,430 CDS, and database searching matched the CDS into 28 categories (**Table 1**). Secreted proteins accounted for 18.7% and 38.8% of the coding sequences and mapped reads, respectively, and included enzymes, protease inhibitors, lipocalins, and immune-related genes. Coding sequences classified with immune functions represented 0.86% and 2.45% of the total CDS and reads, respectively. More than 30% of coding sequences and 3.1% of total reads represented sequences of unknown function. Sorting of significantly expressed coding sequences with their respective reads identified 2,859 differentially expressed CDS between *R. parkeri-*infected and uninfected hemocytes (**Supplementary Table S3**). Of these, 938 belonged to the secreted class, 46 to the immunity class, 568 were unknown, and 48 were associated with cytoskeletal functions. Forty bacterial-derived coding sequences were also significantly differentially expressed between *R. parkeri-*infected and uninfected hemocytes. In addition, we identified 39 coding sequences with functions as regulators of hematopoiesis, hemocyte differentiation, and immune functions. Of the 39, 14 (30.8%) were significantly differentially regulated between *R. parkeri-*infected and uninfected hemocytes (**Table 2**), and these genes include *transglutaminases*, *astakines*, *hemocytin*, *prokineticin, thymosin* and the two *Drosophila* homologs *eater*, *nimrod B2*, Runt-related transcription factor (*Runx*) and GATA-binding factor (*GATA*). We also identified coding sequences in the Toll and immune deficiency (IMD) immune pathways.

**Table 1:** Functional categories of all differentially expressed coding sequences.

Class	Class	Number of CDS	Percent of CDS	Number of Reads	Percent of Reads
uk	Unknown	11870	30.10474524	9603035.74	3.138631215
s	Secreted	7358	18.66139136	118828691.8	38.83766045
te	Transposable element	2885	7.316949453	2672948.89	0.87361966
uc	Unknown, conserved	2325	5.896675036	42390319.04	13.85474158
st	Signal transduction	2002	5.077481042	9833046.42	3.213807307
tm	Transcription machinery	1961	4.973496665	11671193.57	3.814582539
pe	Protein export	1230	3.119531309	8288525.8	2.709000207
tr	Transporters/storage	875	2.219178777	4005011.26	1.30898746
ps	Protein synthesis	826	2.094904766	35877305.11	11.72604505
bac	Bacterial	800	2.028963453	1206145.12	0.394213333
pm	Protein modification	774	1.963022141	7113918.29	2.325094544
nr	Nuclear regulation	762	1.932587689	3512643.3	1.148063197
met/energy	Metabolism, energy	748	1.897080829	6772487.49	2.21350219
cs	Cytoskeletal	683	1.732227548	9305463.74	3.041373557
met/lipid	Metabolism, lipid	679	1.722082731	3831681.35	1.252336763
prot	Proteasome machinery	679	1.722082731	4222526.26	1.380079496
extmat	Extracellular matrix	558	1.415202009	4479967.54	1.464220934
met/carb	Metabolism, carbohydrate	421	1.067742017	3411249.59	1.11492394
detox	Detoxification	419	1.062669609	2614503.07	0.854517381
met/aa	Metabolism, amino acid	384	0.973902458	4133084.08	1.350846447
imm	Immunity	340	0.862309468	7502000.5	2.451934324
met/nuc	Metabolism, nucleotide	281	0.712673413	1291648.15	0.422158921
met/int	Metabolism, intermediate	191	0.484415024	995312.41	0.325305318
tf	Transcription factor	166	0.421009917	789597.62	0.258070032
ne	Nuclear export	77	0.195287732	530713.8	0.173457118
storage	Storage	69	0.174998098	1009052.91	0.329796228
protinhib	Protein inhibition	46	0.116665399	5481.05	0.001791412
vir	Viral	10	0.025362043	18030.95	0.005893189

**Table 2:** Differentially expressed transcripts of hematopoietic, hemocyte functions and immunity rekated genes in Am. maculatum hemocytes with and without R. parkeri.

Comments	Link to PEP	CLSF1 READS	CLSF2 READS	CLSF3 READS	CLFF1 READS	CLFF2 READS	CLFF3 READS	INSF1 READS	INSF2 READS	INSF3 READS	INFF1 READS	INFF2 READS	INFF3 READS	IvsC-cut logFC
Runt1	AmHem-441656	0	0	0	0	3.65	0	28.78	37.15	10.8	30	377.35	10.79	6.716169
Runt2	AmHem-441653	0	0	0	0	3.65	0	28.78	0	0	90	125.78	10.79	5.716385
GATA1	AmHem-289270	0	0	125	74	26	75	79	661	282	269	165	180	5.93738
GATA2	AmHem-210536	126.01	195.01	58.05	45	52.01	49	54	16	11	18	21	36	-2.71693
GATA3	AmHem-273849	194	98	130	99	281	298	178	77	66	101	73	70	-1.93305
GATA4	AmHem-239125	12	0	214	13	45	85	168	268	360	280	238	143	2.77226
GATA5	AmHem-347209	0	0	48.09	15.26	0	0	91.05	623.77	352.16	488.9	212.63	0	7.592625
GATA6	AmHem-347211	64	0	776.46	118.74	80.77	201.21	195.45	1446.23	993.84	897	840.89	353.52	2.407145
GATA7	AmHem-482569	0	6.1	295.25	34.58	95.11	71.03	204.51	494.94	329.54	237.67	91.05	273.38	3.07439
GATA8	AmHem-482570	87.96	20.14	1601.83	266.21	198.81	464.75	732.71	5216.33	3682.94	3206.47	2276.99	751.15	3.114698
GATA9	AmHem-470445	0	0	14.08	11	8	22	0	71.25	31.77	66.58	114.77	6	4.462723
TGASE1	AmHem-380205	101	113	60	71	0	10	3	18	0	0	8	0	-4.87304
TGASE2	AmHem-552537	70	73	40	98	30	2	0	4	0	26	8	0	-5.16345
TGASE3	AmHem-482635	0	0	209	0	42	43.83	65	168.92	247.76	102.75	133	55.86	5.507495
TGASE4	Amac-hemSigP-334835	0	0	29.93	24	0	31	22	93	56	112.55	38	55	4.702866
TGASE5	AmHem-459192	17516.31	46580	14798.9	49356.53	20010.57	17523.8	23585.06	16475	15931	18342.91	12138.12	12258	-2.05595
TGASE6	AmHem-174366	215	207	803	4301	2276	905	211	84	419	1435	566	197	-2.04591
Astakine	AmHem-345670	482	28	8779	675	1866	8381	7415	21497	14407	9849	8468	5215	2.417017
B-Thymosin1	AmHem-338118	1269.22	4245.18	318.8	527.8	398.15	867.17	0	3.12	14.54	0	0	0	-11.5618
B-Thymosin2	AmHem-338117	3654.54	9686.86	6454.94	13506.88	6456.34	5943.88	7390.74	7560.65	7364.46	7809.84	5696.13	2885.93	-1.44851
B-Thymosin3	AmHem-338116	17683.24	34411.97	13377.27	36835.32	18466.52	15594.95	21886.26	13927.22	13598	14227.16	10814.87	11372.07	-1.93252
Laminin1	AmHem-503628	0	0	0	0	0	6	8	87	33	21	42	16	5.488102
Laminin2	AmHem-458927	258	64	4041	349	552	1843	2136	6471	6522	6539	2189	1734	2.314672
Laminin3	AmHem-442680	436.27	64	6474.66	760	957.91	2684.55	3564.65	12770.17	11715.61	10649.03	6290.86	3099.56	2.566915
Laminin4	AmHem-294164	105	68	2535	251	138	500	520	2384	4121	2798	3033	689	2.625331
Laminin5	AmHem-299208	12	0	93.57	0	28	46	35.38	624.97	264.13	277.24	160	163.69	3.239217
Laminin6	AmHem-330549	18.64	0	496	9.48	0	146.02	255.71	911.86	719.29	794.46	408	76.02	3.447777
Laminin7	AmHem-335070	768.68	355.84	357.07	200.41	612.93	332.07	267.53	285.03	129.07	281.61	107.4	28.1	-2.50297
Laminin8	AmHem-335081	0	0	0	0	0	0	111.19	0	0	0	9.06	184.23	8.623004
Laminin9	AmHem-335076	0	0	807.15	0	0	0.01	0	908.95	558.08	730.81	345.28	0	11.12734
Laminin10	AmHem-498018	941.96	673	1101.55	737.61	1234.59	587.27	532.91	981.17	530.21	1009.23	617.14	510	-1.3971
Laminin11	AmHem-419185	6.37	0	818.73	88.42	23.79	116.98	189.05	2619.78	1542.25	1709.65	1684.04	448.15	5.209258
Laminin12	AmHem-419189	154.66	40	1844.74	513.97	487	387.47	593.06	3030.19	2248.51	2263.38	2282.64	476.67	1.869625
Laminin13	AmHem-309325	20	6	358.36	126.45	58.49	146	114.92	969.07	554.61	499.48	321.91	179.15	2.265047
Laminin14	AmHem-338553	0	0	99	22	23.44	14.05	27.03	506.07	278.44	305.03	114.66	74	6.321822
Laminin15	AmHem-374738	38	33	172	4	20	51	40	817	302	335	83	170	2.045175
Laminin16	AmHem-353252	0	0	75.27	0	32.2	24.99	34.58	150.82	137.88	131.35	72.87	0	5.156663
Laminin17	AmHem-346108	34	31	427	58	40	145	210	1598	838	829	389	219	2.451823
Laminin18	AmHem-424284	2312	2245	277590	147937	15396	40352	18474	376831	364739	250183	578039	52921	3.127665
Laminin19	AmHem-497541	830.19	1965.07	1024.26	2165.28	1048.21	680.21	867.73	1493.26	1279.61	1483.25	1155.24	632.23	-1.46151
Laminin20	AmHem-394463	166	16	1083.07	383	252.51	470.47	349.12	1812	1843	1703	1386.17	654	1.626003
Laminin21	AmHem-500241	58	20	661	102	152	216	300	1888	1049	1329	1172	470	2.550937
Laminin22	AmHem-400872	0	0	731.88	166	329.99	69.71	69.7	1425.22	1249.42	862.76	506.54	383.34	6.530802
Laminin23	AmHem-319810	0	0	60.72	0	15.72	187.16	32.18	106.3	187.85	186.11	142.91	263.22	5.185021
Laminin24	AmHem-476724	0	0	212.2	0	0	20.09	0	115.59	376.12	51.76	332.53	145.38	7.035189
Laminin25	AmHem-507361	24	44	852	84	69	163	257	1957	1251	1451	924	290	2.845992
Laminin26	AmHem-349303	13.04	0	86.27	1.93	28.04	36.89	45	143.7	240.46	191.93	50.59	248.3	2.621319
Laminin27	AmHem-285308	374.55	21.29	10641.12	1511.97	833.16	6122.05	2308.26	24848.77	21309.25	15599.81	14990.59	1915.09	2.982538
Laminin28	AmHem-430660	55.85	46.28	322.8	51.03	70.01	103.41	189.86	938	607.59	578	320	88	1.604103
Laminin29	AmHem-187012	22	0	257	44	79	0	0	0	0	0	0	0	-8.17965
Laminin30	AmHem-168121	0	0	28.32	14	14.15	13	0	170.17	93	80.17	56	0	4.826841
Laminin31	AmHem-236364	0	0	140	10	18	34	45	479	330	251	112	19	6.199449
Laminin32	AmHem-128067	15.49	0	0	21.91	0	75	0	0	0	0	0	0	-7.66793
Laminin33	AmHem-413389	138	22	1097	131	396	992	591	3666	2267	3069	1367	458	2.068053
Laminin34	AmHem-505389	92	35	1301.38	127.18	175.21	255.45	412	2265.32	2121.47	2522.58	1563.08	352.65	2.614979
CLIP1	AmHem-164283	0	0	3.18	0	0	0	0	61.06	2	12.62	2.55	0	5.803822
CLIP2	Amac-hemSigP-457530	0	0	0	0	0	0	0	4507.28	4139.48	3022.35	5314.08	0	13.96764
CLIP3	AmHem-462558	572.17	166	5564.33	3103.15	1700.54	5257.31	5943.08	11165.25	10310.12	7719.33	7912.2	2648.68	1.612822
CLIP4	AmHem-462556	71.83	0	322.67	59.85	155.46	403.69	535.92	1121.75	910.88	752.29	834.8	498.32	2.125365
PGRP1	AmHem-345000	89	189	38	115	83	59	34	25	13	36	17	126	-2.55
PGRP2	AmHem-459642	359	337	6	14	0	0	0	1	0	0	0	0	-8.50
PGRP3	Amac-hemSigP-212567	76.56	993.75	53.99	0.03	656.02	168.96	0	0	139.7	0	0	0	-8.32
PGRP4	Amac-hemSigP-370787	245	234	0	4	0	0	10	6	0	0	0	0	-5.46
PGRP5	AmHem-140793	8.96	65.26	3.94	39.45	13.16	9.13	15.51	3	2	2	0	17.08	-3.01
PGRP6	AmHem-429218	16	23	803	112	171	306	238	1372	1238	1185	732	582	2.67
PGRP7	AmHem-310141	83	92	2	2	42	22	0	11	1	0	0	0	-6.93
PGRP8	AmHem-308618	169	256	175	685	317.58	9	0	9	0	0	0	0	-9.90
GNBP1														
Spaetzle1	AmHem-476392	0	0	217.64	38	70.79	12.12	135.33	465	343	223	367	46.61	6.19
Spaetzle2	AmHem-475327	0	32	41	18	6	14	345	53	152	34	336	242	2.76
TOLL1	AmHem-352197	3.18	0	292.06	15.23	20	51.99	224.07	650.07	306.69	89.21	178.68	115.8	3.95
TOLL2	AmHem-450801	75	14	634	69	195	340	569	512	1135	827	697	896	2.01
Myd88	AmHem-151635	0	0	0	0	0	0	0	4	1	0	0	36	NIL
Tube														
Pelle														
Cactus	AmHem-417516	18.71	3.78	656.16	80	93.51	255.81	188.8	1869.25	1031.99	1157.52	794.82	245.58	3.25
Dorsal	AmHem-314664	13	67	444.54	81	21	127	80.9	907	689	493.4	327	227	1.75
Diff	Amac-hemSigP-143049	266	294.73	0	0	11	0	0	0	0	0	0	13	-6.18
Defensin1	Amac-hemSigP-382382	4902	9329	4484.83	9515.23	8557	5643	5865.3	3731.27	3165.44	4720.2	2766.11	2861	-2.07
Defensin2	AmHem-345595	831	981.87	32	26	0	0	4	0	0	0	0	0	-8.64
Defensin3	AmHem-286925	672	872	15	0	2	0	0	1	0	0	0	7	-5.46
Defensin4	Amac-hemSigP-347294	612	264	0	6	0	0	0	2	0	0	0	0	-7.68
Defensin5	Amac-hemSigP-433534	11	26	115	172	181	268	751	110	1021	480	523	274	1.99
Defensin6	AmHem-296786	7767.85	9549.37	133.51	29	15.03	13	31.61	21	0	0	0	24.32	-6.01
Defensin7	Amac-hemSigP-284010	456.67	167.57	685.54	514.87	784.28	728.76	293.29	409.29	307.98	241.5	220.42	664.64	-1.39
Defensin8	AmHem-314023	220	365	30	66	206	23	0	0	0	0	0	0	-11.18
Defensin9	AmHem-205330	85.95	100	186.99	633.91	207.49	205	9	34	18	3	5	30	-4.62
Defensin10	AmHem-482396	622	1077	910	4355	9598	418	0	4	1	0	0	0	-13.10
PGRP1	AmHem-345000	89	189	38	115	83	59	34	25	13	36	17	126	-2.55
PGRP2	AmHem-459642	359	337	6	14	0	0	0	1	0	0	0	0	-8.50
PGRP3	Amac-hemSigP-212567	76.56	993.75	53.99	0.03	656.02	168.96	0	0	139.7	0	0	0	-8.32
PGRP4	Amac-hemSigP-370787	245	234	0	4	0	0	10	6	0	0	0	0	-5.46
PGRP5	AmHem-140793	8.96	65.26	3.94	39.45	13.16	9.13	15.51	3	2	2	0	17.08	-3.01
PGRP6	AmHem-429218	16	23	803	112	171	306	238	1372	1238	1185	732	582	2.67
PGRP7	AmHem-310141	83	92	2	2	42	22	0	11	1	0	0	0	-6.93
PGRP8	AmHem-308618	169	256	175	685	317.58	9	0	9	0	0	0	0	-9.90
Caudal	AmHem-430598	0	28.7	0	10	50.63	1.86	0	0	0	173.33	0	0	-8.01
IAP-2	AmHem-441380	195.12	73	1935.13	556.19	588.52	1079.76	899.76	5189	3745.68	4842.61	2989	940.46	2.09
TAK-1/MAPK-7	AmHem-188995	112	0	1641	181	188.99	388	554	6230	3676	3437	2520	765	3.36
	AmHem-402265	28.3	0	474	111.99	57	150.9	120	1410	681	842	513.23	174.91	2.75
	AmHem-490502	38	0	172	39.96	66	61	115.14	402.97	252.51	364	224	267	1.93
	AmHem-481093	14	30	274	56	48	71	79	581	587	700	369	240	2.33
	AmHem-483742	8	15.57	163.17	36	7.78	83	31.92	733.24	449.45	511.51	214.73	194.61	2.74
	AmHem-452393	753.23	507.11	382.59	412.07	375.32	77.2	0	2	0	0	0	0	-11.42
	AmHem-428323	24	20	88	14	30	60	41	347	283	386	167	169	1.90
TAB-2/MAP3K7IP2	AmHem-223720	8.96	0	90.25	15	46.65	19.75	14	27.79	34.34	19.43	21.53	0	NIL
	AmHem-385445	250.69	0	65.64	266.86	0	0	309.47	27.46	11.42	17.77	17.05	0	
	AmHem-385447	323.49	527	479.34	165.14	484.42	504	184.52	1228.46	914.58	782.31	841.95	494	
	AmHem-385444	10.82	0	11.01	0	0	0	9.01	0	0	3.93	0	0	
IKK gamma	AmHem-322189	121	11	487	70	140	227	308	872	564	514	335	257	NIL
IKK alpha	AmHem-322189	121	11	487	70	140	227	308	872	564	514	335	257	NIL
IKK beta	Amac-hemSigP-43945	0	7	20	0	0	3	0	0	0	2	0	0	NIL
Relish	Amac-hemSigP-143049	266	294.73	0	0	11	0	0	0	0	0	0	13	-6.18
alpha-2-macroglobulin	AmHem-43749	68	384	0	9	0	0	0	0	0	0	0	0	-8.65006
alpha-2-macroglobulin	AmHem-473966	4238	4091	129571	136639	28165	44289	46705	285018	197225	258183	368925	36740	2.228224
alpha-2-macroglobulin	AmHem-340857	135	22	5223	1051	1362	1768	1912	6660	8085	7376	9680	2886	3.048701
alpha-2-macroglobulin	AmHem-241896	6	0	0	31	7	7	33	171	57	124	38	100	2.440071
Complement component C2/Bf precursor	AmHem-459726	202	497	201	34	806	786	42	215	15	502	26	34	-2.97
Eater	AmHem-270031	379	509.19	6	35	0	0	0	0	0	0	1	0	-9.26
FBG, Fibrinogen-related domains (FReDs).	Amac-hemSigP-444130	110.66	92.43	36.77	142.87	84.56	75.24	97.68	47.31	26.31	19.9	18.12	5.97	-2.89
FBG, Fibrinogen-related domains (FReDs).	Amac-hemSigP-470263	0	0	45.94	12.39	4.3	18.18	43.64	117.28	112.15	75.1	56.32	99.46	5.54
FBG, Fibrinogen-related domains (FReDs).	AmHem-396331	2473.98	5610.93	1268.43	2941.73	3165.16	3754.48	2748.24	844.68	1812.83	2360.67	1368.62	1209.84	-2.14
FBG, Fibrinogen-related domains (FReDs).	AmHem-396326	53.65	319.38	68.95	129.76	55.83	111.76	31.3	31.19	85.66	72.05	41.48	0	-2.91
FBG, Fibrinogen-related domains (FReDs).	AmHem-487866	110	853	543	1066	4340	645	0	2	0	1	0	0	-11.80
FBG, Fibrinogen-related domains (FReDs).	AmHem-477539	0	141	0	24.63	289.94	0	0	0	0	0	0	0	-10.19
FBG, Fibrinogen-related domains (FReDs).	AmHem-477540	141	0	393	405.37	1380.06	217	3	0	0	0	0	0	-9.93
FBG, Fibrinogen-related domains (FReDs).	AmHem-376633	57.37	0	107.32	56.57	128.54	7.28	0	5.14	0	0	0	0	-7.51
FBG, Fibrinogen-related domains (FReDs).	AmHem-337488	162	78	17336	3350	3968	3673	5876	13770	12335	8191	18833	4010	2.84
FBG, Fibrinogen-related domains (FReDs).	AmHem-376632	13.63	18	67.68	28.43	88.11	72.4	0	3.86	1	17.31	0	0	-4.57
FBG, Fibrinogen-related domains (FReDs).	Amac-hemSigP-396114	360	113	418	1285	4252	35	0	2	0	0	0	0	-12.01
FBG, Fibrinogen-related domains (FReDs).	AmHem-199732	45.69	70.21	53.81	14.29	58.98	68.73	0	18.4	14.05	7.4	3.51	63.12	-2.71
FBG, Fibrinogen-related domains (FReDs).	AmHem-295667	0	0	18	0	4	6	9	30	63	43	30	0	4.84
FBG, Fibrinogen-related domains (FReDs).	Amac-hemSigP-336380	1767.01	1433.02	890.63	1401.02	1659.63	1118	1284	1021	697	923.87	594	1288	-1.67
Nimrod B2	AmHem-305744	1441.1	3072.34	68.2	491.21	10	20	11.44	80.22	0	0	0	0	-9.52
C-type lectin	AmHem-310057	22.58	89.36	20.87	346.09	25.32	0	0	0	0	0	0	0	-10.31
C-type lectin	AmHem-310058	9.42	275.64	45.13	314.91	28.68	0	0	0	0	0	0	0	-10.94
putative cdc42-interacting protein cip4	AmHem-271438	39	0	652.6	106	45.86	91.67	285	2465.42	1659.98	2179	1041	322	3.78
putative cdc42-interacting protein cip4	AmHem-354954	0	44	269.4	0	66.14	174.33	0	48.58	29.02	0	0	0	-5.54
putative elmo domain-containing protein 1	AmHem-333093	14	14	127	6	24	49	70	208	212	172	76	60	1.62
putative gnlcdd229182 (protein kinase)	AmHem-434547	136	16	745	141	409	498	466	1410	897	1135	663	808	1.37
putative g-protein coupled receptor - signalP detected	Amac-hemSigP-343286	157.35	36	3219.04	1260.82	647.37	1406.01	743.54	12335.87	4744.6	6197.74	5773.28	1567.83	2.75
Rac GTPase-activating protein 1	AmHem-452370	136.29	48	2178.79	307.28	476.07	1026	927.04	6311.96	3527.29	3697.11	2368.85	1339.39	2.50
putative myosin	AmHem-387279	208	16	3912	556	629	798	1887	5248	5861	5978	5633	1497	2.84
putative myosin	AmHem-378134	179	53	2181	568	426	1198	1089	3505	6803	4079	2809	1150	2.28
putative myosin	AmHem-437487	269	37	2512	541	772	1571	1960	6575	8410	7312	6511	2148	2.70
putative protein	AmHem-433409	0	0	29.38	0	0	8.1	18	114.11	64.34	49.91	21.91	51.19	5.93
putative protein - 41 OH-glycosylation sites - 41 NetOglyc sites	Amac-hemSigP-447586	40	14	439	96	84	109	123	1389	881	1169	687	328	2.66
putative protein kinase - GPI anchored	AmHem-299128	793.35	2780.23	1205.82	3614.54	1288.5	1537.72	1997.45	1616.04	1063.19	1032.49	1521.84	1661.27	-1.51
putative ras family	AmHem-328792	24.5	0	227	18.38	42	51	100	411.45	408	542.44	338.49	102	2.73
putative ras subfamily protein of ras small gtpase	AmHem-438000	209	68	3271.28	399.11	1184	1429.96	1663.43	4675.65	5302.59	3922.63	3784.9	892	2.03
putative rho guanine nucleotide exchange factor vav3	AmHem-177587	33	0	571.6	113.51	74.03	257.2	128.48	2604.58	1306.67	1551.51	953.99	386.05	3.32
putative signaling protein (Dedicator of cytokinesis protein 1)	AmHem-340140	217.92	107.24	2279.3	297.45	406.78	884.72	1213.25	2802	2526.33	1998.58	1528.23	1232.65	1.56
putative sphingomyelinase	AmHem-415278	5	37	19	0	85	1	0	2	0	0	0	0	-7.27
putative sphingomyelinase	AmHem-443201	355.15	367.4	69.5	1099.35	630.12	296.54	527.31	16.5	10.04	11.77	37.65	0	-4.15
putative sphingomyelinase - signalP detected	Amac-hemSigP-354535	446	159	15.67	28	183.06	0	0	0	0	0	0	0	-10.92
Scavenger Receptor	AmHem-369012	357	441	6	4	0	0	0	0	0	0	0	0	-8.44
Scavenger Receptor	AmHem-320301	1897	50	158	328	40	38	16	3	0	0	16	0	-6.95
Scavenger Receptor	AmHem-358999	246	871.58	1	0	3	0	0	0	0	0	0	0	-8.38
Scavenger Receptor	AmHem-441843	350.72	144.03	0	0	0	1	0	0	0	0	0	0	-7.07
Scavenger Receptor	AmHem-207626	974.82	1784.14	13.99	0	2	0	0	1	0	0	0	0	-8.00
Scavenger Receptor	AmHem-289376	2292.36	5690.86	24.01	0	1	2	2	6	0	0	0	6	-5.74
Thioester-containing protein	AmHem-349981	0.01	0	100.26	0.27	0.13	104	2.02	146	251.26	313.31	121.45	0.28	5.36
Thioester-containing protein	AmHem-349977	11.99	0	30.74	15.73	19.87	0	90.98	0	35.74	114.69	92.55	385.72	Nil

### 3.6 Regulators of hematopoiesis and hemocyte differentiation

#### 3.6.1 GATA factors and runt domain-containing sequences

Seven genes regulating hemocyte production and differentiation were identified. Two transcription factors, *GATA* (AmHem-289270, AmHem-210536, AmHem-273849, AmHem-239125, AmHem-347209, AmHem-347211, AmHem-482569, AmHem-482570, and AmHem-470445) and *Runt* (AmHem-441656 and AmHem-441653) were differentially regulated between infected and uninfected hemocytes (**Supplementary Figure S4A**). Seven of the nine GATA transcription factors and all the Runt domain-containing sequences were significantly upregulated in *R. parkeri-*infected hemocytes. GATA factors and Runt proteins have previously been shown to be critical in maintaining pluripotent hemocyte precursors in the hematopoietic organ (23, 63).

#### 3.6.2 Astakines, β-thymosin, and transglutaminases

We identified one *astakine* (AmHem-345670) and three *β-thymosin* (AmHem-338118, AmHem-338117, and AmHem-338116) coding sequences differentially regulated upon *R. parkeri* infection (**Supplementary Figure S4A**). *R. parkeri* led to a three-fold upregulation of the AmHem-345670 transcript (*astakine*) and downregulation of the three *β-thymosin* transcripts (AmHem-338118, 11-fold; AmHem-338117, 2-fold; and AmHem-338116, 2-fold). *Astakines* are ancient cytokines with conserved cysteine domains that share similar homology to vertebrate prokineticins (64, 65). *β-thymosins* are small peptides involved in numerous cellular processes such as cellular migration, tissue repair and cell adhesion, proliferation, and differentiation in vertebrates. Their affinity for ATP-synthase is crucial to their function (66). Like *β-thymosins*, *transglutaminases* (TGases) are ubiquitously expressed and regulate many cellular processes such as cellular adhesion, cell migration, and the maintenance of the extracellular matrix. Six TGases (AmHem-380205, AmHem-552537, AmHem-482635, Amac-hemSigP-334835, AmHem-459192, and AmHem-174366) were significantly upregulated, except for AmHem-482635 and Amac-hemSigP-334835, which were downregulated on *R. parkeri* infection (**Supplementary Figure S4A**).

#### 3.6.3 Laminin receptors and CLIP-domain serine proteases

Laminin receptors are a group of proteins with diverse biological functions, including cellular differentiation. They serve as binding partners with different homeostasis-associated proteins to maintain hemocyte homeostasis (67). The *CLIP*-domain serine protease (*CLIPsp*) is abundantly present in the hemolymph of insects and arthropods. Four transcripts with a Clip or disulfide knot domain (AmHem-164283, Amac-hemSigP-457530, AmHem-462558, and AmHem-462556) were differentially expressed in our dataset (**Supplementary Figure S4A**): *R. parkeri* led to six-fold upregulation of AmHem-AmHem-164283, 14-fold upregulation of Amac-hemSigP-457539, and two-fold upregulation of AmHem-462558 and AmHem-462556. In invertebrates, these proteins play dual roles in innate immune responses and hematopoiesis, acting as binding partners of *toll*-like receptor *Spaetzle*, leading to downstream transcriptional activation of antimicrobial peptides. Similarly, they activate the prophenoloxidase (PPO) cascade necessary for melanization (68). A direct role has been described for *CLIPsp*-induced PPO maintenance of hematopoiesis (64).

### 3.7 Regulators of hemocyte-mediated cellular functions

Hemocyte-mediated cellular responses are an important component of the invertebrate innate immune system, and hemocyte functions, such as phagocytosis, are relatively conserved across invertebrate species. Several cell surface receptors are involved in the cellular immune response. In *Drosophila* and mosquitoes, the eater and nimrod transmembrane receptor families of proteins serve as phagocytosis receptors and scavenge bacteria for phagocytic killing. AmHem-270031 (homolog of *eater*) and AmHem-305744 (homolog of *nimrod B2*) were significantly downregulated (>9-fold) in *R. parkeri-*infected hemocytes (**Supplementary Figure S4B**). Thioester-containing proteins (TEPs) are like the mammalian complement system and are involved in microbial opsonization prior to phagocytosis. We found three TEPs in our transcriptome data, with all transcripts (AmHem-349981, AmHem-459726, and AmHem-349977) containing an alpha-2-macroglobulin domain (**Supplementary Figure S4B**). AmHem-349981 and AmHem-349977 were 5-fold upregulated, while AmHem-459726 was 3-fold downregulated in *R. parkeri-*infected hemocytes. Four alpha-2-macroglobulin (α2-macroglobulin) transcripts (AmHem-43749, AmHem-473966, AmHem-340857, and AmHem-241896), each consisting of the complement component region of the alpha-2-macroglobulin family, were also differentially expressed (**Supplementary Figure S4B**). Three of the four α2-macroglobulin transcripts (AmHem-473966, AmHem-340857, and AmHem-241896) were >3-fold upregulated, while AmHem-43749 was 9-fold downregulated in *R. parkeri-*infected hemocytes. However, AmHem-43749 was only expressed in two of the six uninfected hemocyte groups.

AmHem-369012, AmHem-320301, AmHem-358999, AmHem-441843, AmHem-207626, and AmHem-289376 were transcripts containing secretory signal peptides with class F scavenger receptor domains, and all were significantly downregulated >6-fold in *R. parkeri-*infected hemocytes. Fourteen transcripts (Amac-hemSigP-444130, Amac-hemSigP-470263, AmHem-396331, AmHem-396326, AmHem-487866, AmHem-477539, AmHem-477540, AmHem-376633, AmHem-337488, AmHem-376632, Amac-hemSigP-396114, AmHem-199732, AmHem-295667, and Amac-hemSigP-336380) containing fibrinogen-related domains (FReDs) were significantly downregulated in *R. parkeri-*infected ticks (**Supplementary Figure S4B**). FReD-containing proteins are involved in complement activation and phagocytosis in mammals (69), and several have been identified in invertebrates such as crabs (70), snails (71), mosquitoes (72, 73), and ticks (62, 74). Our data also showed significant downregulation (>10-fold) of two transcripts (AmHem-310057 and AmHem-310058) with a lectin C-type domain and mannose-binding activity. The binding activities of lectins make them suitable for pathogen recognition and are an important component of the immune response.

### 3.8 Regulators of the toll pathway

**Figure 6:**
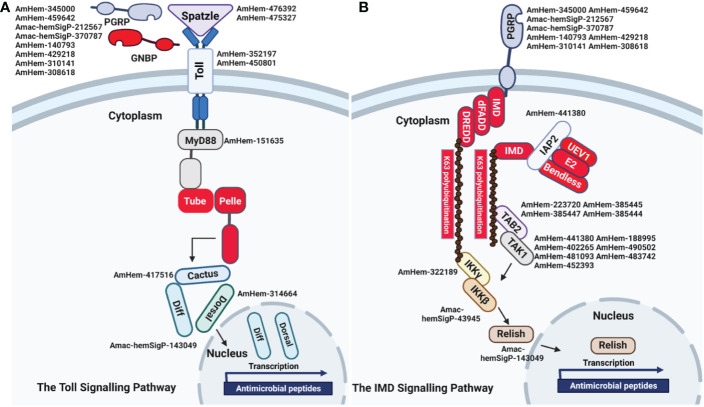
Representative signaling pathways and immune-related genes. Reconstruction of the immune-signaling genes derived from *R. parkeri-*infected and uninfected hemocytes showing the components of the **(A)** Toll and **(B)** IMD signaling pathways. The Toll and IMD signaling pathways are highly conserved in ticks. Transcripts highlighted in red were not identified in this study.

The Toll pathway is highly conserved in both insects and other arthropod species. The peptidoglycan recognition receptor proteins (PGRPs) recognize lysine-type peptidoglycan on the cell wall of Gram-positive bacteria. In contrast, recognition of fungal β1-3-glucan occurs *via* the Gram-negative binding proteins (GNBPs) (75, 76). This binding leads to translocation of nuclear factor kappa B (NF-κB) into the nucleus and subsequent upregulation of antimicrobial peptides. Eight PGRP transcripts (AmHem-345000, AmHem-459642, Amac-hemSigP-212567, Amac-hemSigP-370787, AmHem-140793, AmHem-429218, AmHem-310141, and AmHem-308618) were identified in our RNA-seq dataset. Seven of the PGRP transcripts were 3-10-fold downregulated following *R. parkeri* infection, while AmHem-429218 was 3-fold upregulated. AmHem-459642, Amac-hemSigP-370787, and AmHem-308618 are secreted, while AmHem-140793 is the only differentially expressed membrane-bound PGRP transcript in our dataset (**Supplementary Figure S5**). Nine genes encoding Toll-related receptors have been reported in *Drosophila* (77), with some yet to be identified in the tick genome. All the components of the Toll pathway were detected and differentially regulated in our transcriptome data (**Supplementary Figure S5**) except for *GNBP*, *Tube*, and *Pelle*, the latter two gene products forming a heterodimer with MyD88 in *Drosophila* (77). Activation of *Spaetzle*, a ligand for the Toll receptor, is the rate-limiting step leading to activation of the Toll pathway. AmHem-476392 (6-fold upregulated) and AmHem-475327 (2-fold upregulated) were differentially expressed in *R. parkeri-*infected hemocytes (**Supplementary Figure S5**). Two Toll receptors with a leucine-rich repeat ribonuclease inhibitor domain, AmHem-352197 (4-fold upregulated) and AmHem-450801 (2-fold upregulated), were significantly expressed in our dataset (**Figure 6A**; **Supplementary Figure S5**). However, of the MyD88-Tube-Pelle heterotrimeric complex, only one *Myd88* transcript, AmHem-151635 (upregulated), was detected in our dataset. AmHem-417516 (4-fold upregulated), an ankyrin repeat and DHHC-type Zn-finger domain-containing protein encoding Cactus, a negative regulator of the Toll pathway that binds and prevents nuclear translocation of two Rel proteins, *Dorsal* and *Dif* (78), was significantly expressed in our data. We also identified a homolog of *Dorsal*, AmHem-31466 (2-fold upregulated), and *Dif*, Amac-hemSigP-143049 (7-fold downregulated), both containing a Rel homology domain (RHD) of RelA and RelB respectively (**Figure 6A**; **Supplementary Figure S5**). Nuclear translocation of *Dorsal* and *Dif* regulates AMP expression, especially the defensin and drosomycin family of AMPs. From our data, ten *Defensin* transcripts (Amac-hemSigP-382382, AmHem-345595, AmHem-286925, Amac-hemSigP-347294, Amac-hemSigP-433534, AmHem-296786, Amac-hemSigP-284010, AmHem-314023, AmHem-205330, and AmHem-482396) were significantly downregulated in *R. parkeri-*infected hemocytes (**Figure 6A**; **Supplementary Figure S5**).

### 3.9 Regulators of the IMD pathway

The immune deficiency (IMD) pathway is activated upon stimulation of PGRPs by the Gram-negative diaminopimelic acid (DAP)-type peptidoglycan, which stimulates both soluble and transmembrane PGRPs. In contrast to the Toll pathway, the IMD pathway contributes to the production of most AMPs in *Drosophila* (79). In our dataset, we identified the differential regulation of several transcripts in the IMD pathway including inhibitor of apoptosis 2 (*IAP2*), mitogen-activated protein kinase-7 (*MAPK7/TAK1*), mitogen-activated protein kinase 7-interacting protein 2 (*MAP3K7IP2/TAB2*), inhibitor of nuclear factor kappa-B kinase subunits (*IKK*), and *Relish*. AmHem-441380 (2-fold upregulated in *R. parkeri-*infected hemocytes) is an *IAP-2* transcript with baculovirus inhibitor of apoptosis protein repeat (*BIR*), ring finger, and zinc finger domains characteristic of IAP proteins. These proteins regulate NF- κB signaling pathways in the cytoplasm (80). MAPK7/TAK1, TAB-2/MAP3K7IP2, and the IKK complex induce cleavage of Relish and subsequent nuclear translocation by transferring a phosphate group to Relish. We identified seven transcripts of *MAPK7/TAK1* (AmHem-188995, AmHem-402265, AmHem-490502, AmHem-481093, AmHem-483742, AmHem-452393, and AmHem-428323) and four *TAB-2/MAP3K7IP2* transcripts (AmHem-223720, AmHem-385445, AmHem-385447, and AmHem-385444), each with STKc and TyrKc domains, which are the catalytic domain of the serine/threonine kinase and tyrosine kinase catalytic domains, respectively (**Figure 6B**; **Supplementary Figure S6**). Infection with *R. parkeri* upregulated of all the *MAPK7/TAK1* transcripts except for AmHem-452393 (12-fold downregulated). Sequences encoding *IMD*, Fas-associated *via* death domain (*FADD*), and death-related ced-3/Nedd2-like caspase (*DREDD*) genes were absent in our dataset.

### 3.10 Nimrod B2 and eater mediate hemocyte phagocytosis

**Figure 7:**
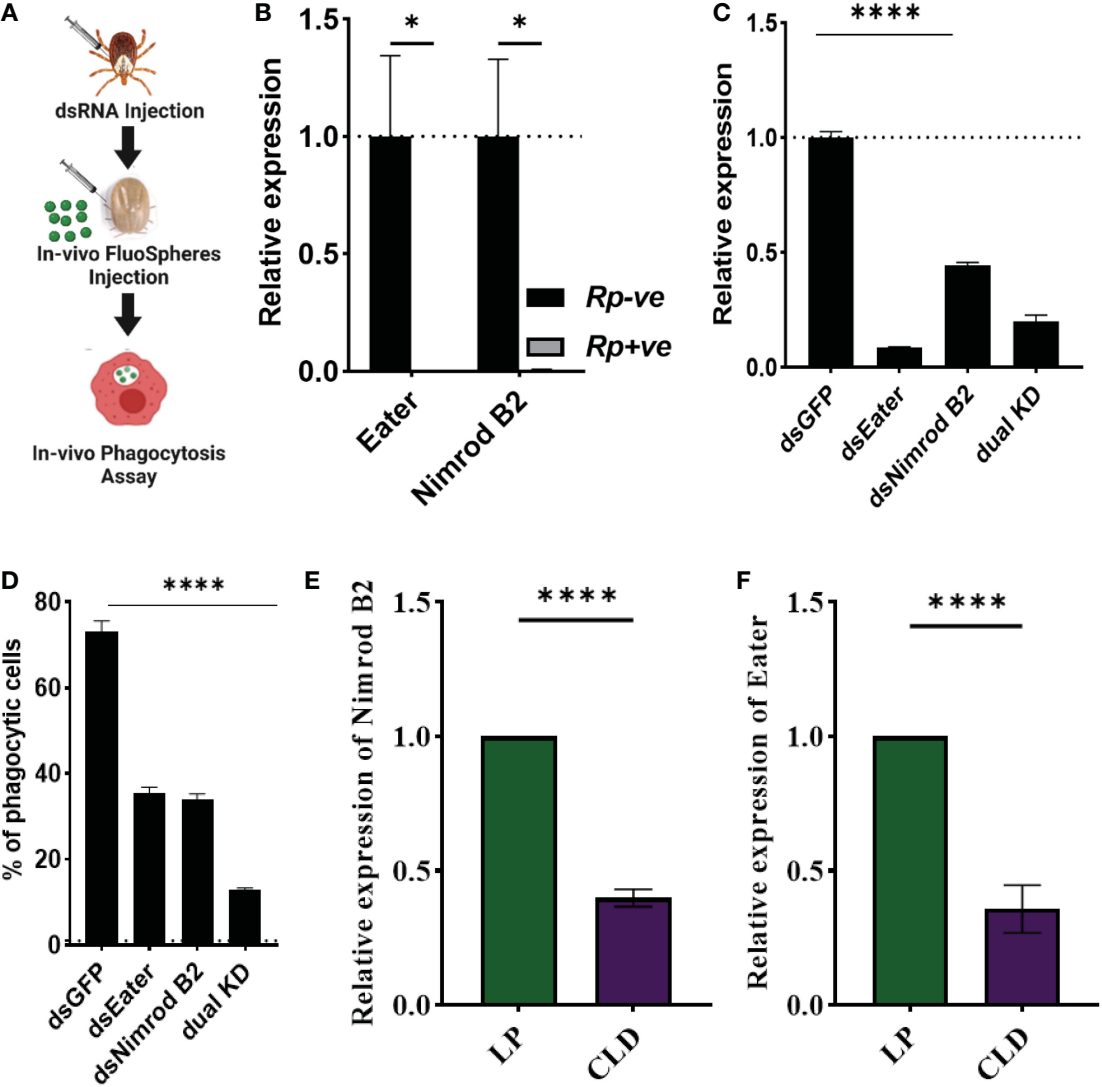
*Nimrod B2* and *eater* homologs as functional markers of hemocyte phagocytosis. The role of *nimrod* B2 and *eater* silencing on *in vivo* phagocytosis was evaluated in *Am. maculatum* hemocytes **(A)**. qPCR validation of bulk RNA expression profiles of *nimrod B2* and *eater* in uninfected and *R. parkeri-*infected *Am. maculatum* hemocytes **(B)**. dsRNA was injected into *Am. maculatum* female ticks to disrupt the expression of *nimrod B2* and *eater* genes and confirmed by qPCR **(C)**. The proportion of phagocytic hemocytes was compared with dsGFP-injected ticks **(D)**. Additional validation of phagocyte depletion showing a significant reduction in *nimrod B2* **(E)** and *eater* **(F)** transcript in CLD-injected ticks. Gene expression was normalized to *Am. maculatum actin.* Data were analyzed using unpaired t-tests in GraphPad Prism v8.4.1. *P < 0.05, ****P < 0.0001.

Hemocytes participate in humoral and cellular defenses in response to microbial infections. Their specific roles are defined by their expressed cell surface receptors. *Nimrod B2* and *eater*, which mediate microbial phagocytosis upon infection (81, 82), were downregulated in hemocytes from *R. parkeri-*infected ticks. Their role in hemocyte phagocytosis and as markers of phagocytic hemocytes have been described in hematophagous and non-hematophagous organisms (81–84). We therefore further defined the role of *nimrod B2* (AmHem-305744; 40% homology to *Drosophila nimrod B2*) and *eater* (AmHem-270031; 32% homology to *Drosophila* eater) homologs in hemocyte phagocytosis using a combination of RNAi and *in vivo* phagocytosis approaches (**Figure 7A**). Quantitative PCR validation of their expression supported the RNA-seq result (**Figure 7B**). Their transcripts were significantly depleted in dsRNA-injected tick hemocytes (**Figure 7C**), and the *in vivo* bead phagocytosis assay also revealed depletion in hemocyte phagocytosis of yellow-green FluoSpheres (beads) (**Figure 7D**). The *Drosophila* homologs, *nimrod* B2 and *eater* were both significantly downregulated in CLD-depleted hemocytes in our phagocytosis assay (**Figures 7E, F**), confirming their specificity to phagocytic hemocytes. Together, these data demonstrate that *nimrod B2* and *eater* are two potential candidate marker genes regulating hemocyte phagocytosis.

## 4 Discussion

Here we report morphological and functional heterogeneity in *Am. maculatum* hemocytes. We define a role for phagocytic hemocytes in the immune response against bacterial infections and identify potential molecular markers of hemocyte phagocytosis. We report for the first time direct evidence of *R. parkeri* infection of phagocytic hemocytes, which might play a role in the dissemination of these organisms throughout the tick body.

Previous studies have identified different hemocyte subtypes in hard (19, 29, 30) and soft (3, 59) tick species, which vary depending on the developmental stage, infection status, and sex. Here we identified five unique hemocyte subtypes based on histomorphological analysis. Female adult ticks pose the most threat to human and animal hosts through hematophagy and pathogen transmission, but we did not detect significant differences between hemocyte populations in unfed male and female ticks. Nevertheless, there were functional differences between male and female ticks, with hemocytes from female ticks displaying more phagocytic hemocytes than those from males. The functional distinction between male and female ticks could be attributed to the presence of more phagocytic plasmatocytes in female than male ticks. More phagocytic hemocytes may also be needed in female hemolymph due to their large body size relative to males, with the prolonged feeding time on the host of females increasing the chance of microbial growth within the tick, thus necessitating a more robust and primed immune system. We however noted that some hemocytes did not stain with Vybrant-CM Dil which could be attributed to the loss of membrane structure that occur during cell death. Immune priming in invertebrates mimics the vertebrate adaptive immune response upon pathogen infection. While immune system priming is well described in mosquito vectors (85–87), only one study has described the presence of active immune priming in ticks (88). We also observed that blood feeding increased granulocytes in female and plasmatocytes in male ticks.

Although granulocytes are professional phagocytic hemocytes, plasmatocytes have also been shown to display phagocytic functions. The decline in the prohemocyte population in blood fed ticks suggests their differentiation to mature form of hemocytes. Prohemocytes are immature hemocytes that can differentiate into other hemocyte types, and their absence would indicate that new hemocytes are produced during feeding, with prohemocytes the source of those new hemocytes. Together, these data for the first time show the heterogeneous nature of the *Am. maculatum* hemocytes and functional differences in male and female hemocytes. Developing specific functional or molecular markers will now be important to confirm our morphological classification.

Chemical inhibitors have been widely used to characterize mammalian immune cells. In the absence of molecular markers to characterize tick hemocytes, hemocytes can be studied and characterized by inhibiting their functions, similar to the widely used reverse genetic approach for gene characterizations. Clodronate liposomes (CLD) are widely used to deplete phagocytic macrophages in mammalian systems (53, 54, 89). CLD mediates phagocyte killing by releasing toxic clodronate upon macrophage phagocytosis, which subsequently induces apoptosis (53). Phagocytic hemocytes have been depleted with CLD in mosquitoes and *Drosophila* (31, 32). We also showed that CLD can successfully reduce the phagocytic hemocyte population in *Am. maculatum*, as evidence by a reduced proportion of phagocytic hemocytes and subsequent loss in functional phagocytosis confirmed by immunofluorescence on CLD treatment. The EdU incorporation assay employed in this study further shows that CLD could also interfere with the replication of hemocyte, however the mechanism behind this process requires further investigation. These findings were further supported by a decrease in transcript levels of *nimrod B2* and *eater* homolgs in CLD-treated ticks, both of which are well characterized phagocyte markers in mosquitoes (32, 82) and *Drosophila* (80, 81, 83). In the tick system, we showed that *nimrod B2* and *eater* homolog knockdown significantly reduced the function of phagocytic hemocytes, further suggesting that these two genes may be useful candidate markers of phagocytic hemocytes in *Am. maculatum*. By showing successful depletion of phagocytic hemocytes using CLD, these experiments provide the means to functionally characterize phagocytic hemocytes in *Am. maculatum* and other tick species. Our experiments also serve as proof of concept for using CLD in the functional study of phagocytic hemocytes in a non-model organism such as ticks. Due to the lack of specific antibodies targeting these two genes, further characterization of their functions is limited.

As the first component of the cellular immune response, phagocytic hemocytes are primarily responsible for scavenging and removing invading pathogens, mainly *via* phagocytosis. The reduced survival of CLD-depleted ticks following bacterial challenge further highlights the critical role of hemocytes in maintaining immune responses in ticks, consistent with immune impairments following CLD depletion seen in mosquitoes and *Drosophila* (31, 32). We show that survival is reduced in CLD-depleted ticks following the *R. parkeri* challenge. Although the pathogenicity of *R. parkeri* to its tick vector is unclear, this result suggests that phagocytic hemocytes have a role in controlling *R. parkeri* infection. While ours is the first functional validation of phagocytic hemocytes by CLD depletion in ticks, two studies used alternative approaches to inhibit hemocyte function and demonstrated reduced survival in ticks following bacterial challenge (6, 11). The outcome of our experiments and similar reports in other organisms highlight the functional conservation of these immune cells in invertebrates.

Hemolymph is a well-defended niche containing hemocytes and several soluble effector molecules that directly inactivate or kill invading microbes (90, 91). Pathogens acquired *via* an infected host’s blood must leave the blood bolus and infect the midgut and resident tissues. The process of *R. parkeri* dissemination from the point of midgut infection to other tick tissues is not fully understood. It has been proposed that rickettsiae can cross the midgut barrier to infect hemocytes during blood feeding (92), and others have also demonstrated *Rickettsia* infection of the tracheal system (93). The constant bathing of tissues with hemolymph and the persistence of the tracheal system through developmental stages make them viable routes for the systemic maintenance and dissemination of rickettsiae throughout the tick body. In the current study, we detected rod-shaped *R. parkeri* in the hemocytes of naturally infected *Am. maculatum.* Likewise, capillary feeding and microinjection of GFP-expressing *R. parkeri* also led to the observation of *Rickettsia* organisms in hemocytes, suggesting active infection of hemocytes by the pathogen. Dissemination of *R. parkeri* to the *Am. maculatum* midgut, salivary gland, and ovarian tissues has previously been described following capillary feeding (94); however, dissemination into the hemolymph and infection of hemocytes is a novel observation that argues for a role for circulating hemocytes in rickettsial trafficking throughout the tick body. Our detection of *R. parkeri* in the hemocytes of infected ticks and tissue dissemination of *R. parkeri* in *Am. maculatum* salivary glands, midguts, and ovaries reported by Harris et al. (94) and (95) strongly suggest that the pathogen survives long enough in the hostile hemolymph environment to find its way to other tick organs. Similarly, the detection of *Rickettsia* Sca2 proteins in unpermeabilized and permeabilized hemocytes likely suggest a bacteria mediated entry into the hemocytes or at the very least a direct interaction between the bacteria and the immune cells. Sca2 is a formin-like protein that is used for adherence, cellular entry and actin-based motility in mammalian host cells, however it is not required for *R. parkeri* infection of the tick vector (94). Infection of tick hemocytes was also shown to enable the migration of *A. phagocytophilum* from the gut to salivary glands of *Ix. scapularis* (22). *R. parkeri* and other vector-borne diseases have evolved a complex process enabling them to colonize and disseminate throughout the arthropod host through transovarial and transstadial transmission (38, 39, 95, 96). Hemocytes, especially phagocytic hemocytes are also required for the dissemination of arboviruses in mosquitoes. Leite at al (61). demonstrated that blocking of phagocytosis in *A. aegypti* prior to feeding on Zika or Dengue virus infected blood lead to a significant decrease in the prevalence of infection. The evidence presented here and previous report by Liu et al. (22) and Leite et al. (61) further argues for a wide array of mechanisms by which *R. parkeri* potentially disseminate through the tick vector. In addition, tracheal infection, and subsequent dissemination from the midgut to the salivary glands have been demonstrated for other rickettsiae as shown with *R. monacensis* in *Ix. scapularis* (93). However, further experiment is required to show direct evidence of *R. parkeri* infection of tick hemocytes and its role in dissemination across tick tissues.

Similar to this, salivary gland colonization during feeding is a crucial step for infecting the mammalian host (97, 98). The presence of sessile hemocytes (tissue-associated hemocytes) has been described in mosquitoes and *Drosophila* (99, 100), and these hemocytes are found in those regions of the body that interact most with invading pathogens such as the periosteal region and abdominal walls (99). However, their role in transovarial and transstadial maintenance of pathogens remains unknown. Whether hemocytes play a role in the trafficking of pathogenic bacteria to tick tissues as observed for mosquito phagocytes and viral infection still needs to be established (61). The mechanisms by which *R. parkeri* enter and survive inside hemocytes also require further examination.

Molecular studies of tick hemocyte biology and hemocyte-mediated immune responses have been limited due to, in part, the technical challenges surrounding hemolymph collection from different tick stages, the lack of a hemocyte-like cell line (as with *Drosophila* and mosquitoes), and a lack of hemocyte-specific markers. Current tick cell lines have been utilized extensively to characterize humoral immune responses which are reflective of changes occurring in the tissue *in-vivo* (101, 102). To identify the molecular responses in *Am. maculatum* hemolymph during infection, hemocyte transcriptomes from *R. parkeri-*infected and uninfected ticks were isolated and analyzed. We identified an entire repertoire of transcripts differentially expressed in hemocytes on *R. parkeri* infection (**Supplementary Table S2**). We identified several genes that mediate the cellular immune response, especially genes encoding hematopoietic functions and hemocyte differentiation. The anatomical structure and molecular basis of hemocyte production in ticks are still unknown. An earlier study on hemolymph circulation proposed the presence of a lymph-like organ as the site of hemolymph production in ticks (13), but the active production of hemocytes from the tick lymph gland or a specialized hematopoietic organ has yet to be demonstrated. The presence of transcriptional and humoral regulators of hematopoiesis genes suggests that the regulation of hematopoiesis is conserved in arthropod and insect species. For instance, the transcription factors *GATA* and *Runt* were detected in our data, which are critical for the proliferation of hematopoietic stem cells and differentiation of myeloid stem cells, respectively, in invertebrates (103). *Lozenge*, a Runt homolog, has been reported to mediate crystal cell maturation in *Drosophila* (103, 104), while in crayfish it mediates differentiation from hematopoietic stem cells to mature granular and semigranular cells (64). Kwon et al. also demonstrated a reduction in oenocytoid differentiation following silencing of *Lozenge* in *An. gambiae* mosquitoes (55) as well as a decrease in the expression of *PPO3/8*, which are molecular markers of oenocytoid cells (55). *Serpent* is a *Drosophila* GATA factor that regulates hematopoiesis and is an ortholog of the vertebrate GATA family (105, 106). *Serpent* is expressed in hemocyte precursors but, unlike *lozenge*, it is still expressed in mature prohemocytes and crystal cells, suggesting roles beyond hematopoiesis (107). No studies have yet identified *Runt* or its homolog in any tick species; however, a GATA factor has been described in *Haemaphysalis longicornis* that activates *Vitellogenin*, which is essential for reproduction (108–110). The presence of transcripts that encode humoral regulators of hematopoiesis, such as *Astakines, β-thymosin*, and *transglutaminases*, was an unexpected finding. Astakines regulate hematopoiesis *via* interaction with transglutaminases or *via* direct interactions with hematopoietic cells to promote structural rearrangements (64). Proteins containing thymosin domains have been described in *Drosophila*, where Ciboulot, a protein with three thymosin domains, regulates axon growth during brain metamorphosis (111). Thypedin, a protein with 27 thymosin domains, regulates foot regeneration in Hydra (112). Ovarian expression of *β-thymosin* was reported in the dog tick, *Dermacentor variabilis* (113). In invertebrates, TGases were initially described as coagulation factors and later shown to regulate hematopoiesis in crustaceans (114, 115) by maintaining hematopoietic cells in an undifferentiated state, thus suppressing hematopoiesis (116, 117). It is currently unclear how these genes regulate the production and differentiation of hemocytes in ticks. However, the presence of some of these genes, such as *Astakines*, in the genomes of crustaceans, ticks, scorpions, spiders, and other arthropods but not *Drosophila* or mosquitoes suggests evolutionary divergence in hematopoietic processes (118).

Several PGRP-encoding transcripts were detected, consistent with a previous hemocyte transcriptome study in *Ixodes scapularis*, where twenty-six PGRP-encoding sequences were detected (62). Genes encoding the Toll and IMD pathways have previously been reported in ticks, although some components of these pathways have yet to be identified (88, 119–123). While many genes encoding Toll and IMD pathway components were identified in our data, it is unsurprising that some Toll and IMD pathway components were not identified in this study. For instance, the absence of *GNBP*, *Tube*, and *Pelle* transcripts - components of the Toll pathway - and the IMD components *IMD*, *FADD*, and *DREDD* is supported by the absence of these transcripts from the *Ix. scapularis* and *Rhipicephalus microplus* genomes (119, 124). The differences observed in the components of the immune pathways from our study when compared to other tick species may be a result of the unique evolutionary relationship that exist between the tick vector and pathogenic microbes they vector or that driven by their resident commensal microbial communities. Shaw and colleagues described plasticity in the immune pathways between insects and arthropods, demonstrating activation of the *Ix. scapularis* IMD pathway by two infection-derived glycerol following pathogen infection (88). Our knowledge of tick immunity has hitherto relied on model organisms like *Drosophila* and mosquitoes, but our data and that from recent studies on tick immunity argue that immunity in hematophagous arthropods such as ticks is very different (88, 119, 124).

Cellular immunity relies on hemocytes directly killing invading microbes *via* phagocytosis, melanization, or the production of reactive oxygen species. Although there have been many studies of cellular immunity in ticks, the roles of specific immune cell types, their molecular signatures, and their contribution to the hemocyte-mediated immune response are still not fully understood. Our study identifies a repertoire of potential candidate genes that regulate hemocyte functions. An unexpected observation was the identification of *nimrod B2* and *eater* transcripts, two *Drosophila* homologs which were significantly expressed in uninfected ticks but downregulated on *R. parkeri* infection. *Nimrod B2* and *eater* are both transmembrane receptors expressed on hemocyte membranes shown to be responsible for the phagocytosis of Gram-positive and Gram-negative bacteria in *Drosophila* (81, 82, 84) and mosquitoes (32, 82, 125). Although the precise roles of *nimrod B2* and *eater* in tick immunity are unknown, our RNAi experiments suggest that they may play a direct role in hemocyte phagocytosis, however the precise mechanisms involved are yet to be clarified. Further studies are now required to determine the cellular localization of these two genes and whether their expression is unique to phagocytic hemocytes from other tick species. Our study also confirms the presence of an active complement component system with several TEPs. The tick complement system is essential for killing tick-transmitted pathogens, as demonstrated by Urbanová and colleagues (4), who demonstrated phagocytosis of the spirochete *Borrelia afzelli* in *Ix. ricinus*. A similar role for tick complement-like proteins in the phagocytosis of non-pathogenic microbes was also demonstrated in *Ix. ricinus* ticks (4, 126, 127).

## 5 Conclusion

Here we describe morphological and functional differences in *Am. maculatum* hemocytes and characterize transcriptional changes in cellular and humoral responses in *R. parkeri* infected and uninfected tick. Our results reveal heterogenous hemocyte populations in *Am. maculatum* showing variable phagocytic capacity. We also describe an integral role for phagocytes in responses to microbial pathogens. We for the first-time observed *R. parkeri* in phagocytic hemocytes. Our transcriptome analysis of *R. parkeri-*infected and uninfected hemocytes allowed us to explore differentially expressed immunity and hematopoietic genes on *R. parkeri* infection. This “big” transcriptome dataset will be important for identifying potential biomarkers of hemocyte subtype, function, and production. Our findings also raise important questions about the role of each hemocyte subtype in immune responses and vector competence.

## Data availability statement

The datasets presented in this study can be found in online repositories. The names of the repository/repositories and accession number(s) can be found below: https://www.ncbi.nlm.nih.gov/genbank/, PRJNA878782 https://www.ncbi.nlm.nih.gov/genbank/, SAMN30755417 & SAMN30755418 https://www.ncbi.nlm.nih.gov/genbank/, GKCB01000001-GKCB01011171.

## Ethics statement

The animal study was reviewed and approved by Protocols for tick blood-feeding were approved by the University of Southern Mississippi’s Institutional Animal Care and Use Committee (USMIACUC protocols #15101501.3 and 17101206.2).

## Author contributions

Conceptualization: AA, RS, SK. Data Curation: AA, JR, SK. Formal analysis: AA, JR, SB, RS, SK. Funding acquisition: SK Investigation: AA, JR, SB, RS, SK. Methodology: AA, JR, RS, SK. Project Administration: SK Resources: RS, SK. Supervision: SK. Writing original draft: AA, SK. Writing, reviewing & editing: AA, JR, SB, RS, SK. All authors contributed to the article and approved the submitted version.

## Supplementary material

Supplementary Figure 1
**(A–C)** Microscopic examination and immunostaining of hemolymph. Light microscopic examination of perfused hemolymph at **(A)** lower and **(B)** higher magnification showing multiple hemocyte populations. **(C)** Immunostaining of perfused hemocytes with WGA lectin (green), Vybrant CM-Dil (red), and Hoechst 33342 stains (blue). Scale bars as indicated. **(D–I)** Hemocyte populations in unfed ticks. Hemolymph was perfused from unfed male and female ticks and the **(D)** total and **(E–I)** differential hemocyte populations compared between unfed and partially fed male and female ticks using an improved Neaubeur chamber. Data were analyzed using unpaired t-tests in GraphPad Prism v8.4.1. *P < 0.05, **P < 0.01, ***P < 0.001, ****P < 0.0001. UF; unfed, PF; partially blood fed.

Supplementary Figure 2
**(A, B)** Confocal images of phagocytic hemocytes infected with *Rickettsia parkeri*. Representative confocal images of immunofluorescence staining for *R. parkeri Sca2* protein in hemolymph of *R. parkeri-*infected *Am. maculatum.* Hemocytes were either **(A)** permeabilized with Triton-X or **(B)** not permeabilized before incubation with *Sca2-*specific antibodies (red) and Hoechst 33342 (blue).

Supplementary Figure 3 LysoTraker Red labeling of lysosomal compartments in ticks fed with *R. parkeri*. Fluorescent microscopic images of hemocytes from *R. parkeri* infected ticks stained with LysoTracker Red (a lysosomal marker) 24 hours after infection. Hemocytes were incubated with primary antibodies against R. parkeri outer membrane protein (green) and Hoechst 33342 (blue). Scale bar = 10 μm.

Supplementary Figure 4
**(A, B)** Heatmaps of RNA-seq expression data showing hematopoietic and cellular function genes differentially regulated in *R. parkeri-*infected compared with uninfected hemocytes. Several of these transcripts include **(A)** transcriptional and **(B)** humoral regulators of hemocyte differentiation and maturation. The scale bar shows the log_2_fold expression value of the transcripts.

Supplementary Figure 5 Heatmap of RNA-seq expression data showing differentially expressed transcripts in the toll-signaling pathway in *R. parkeri* infected and uninfected hemocytes. Six of the nine Toll genes were identified including eight *PGRP* transcripts. Transcripts encoding *GNBP*, *tube*, and *pelle* were, however, not present. The scale bar to the right shows the log_2_fold expression value of the transcripts.

Supplementary Figure 6 Heatmap of RNA-seq expression data showing differentially expressed transcripts in the IMD signaling pathway in *R. parkeri-*infected and uninfected hemocytes. Sequences encoding *IMD*, Fas-associated *via* death domain (*FADD*). and death-related ced-3/Nedd2-like caspase (*DREDD*) genes were absent from our data. The scale bar to the right shows the log_2_fold expression value of the transcripts.
