# Supplementary material for: Rickettsia parkeri hijacks tick hemocytes to manipulate cellular and humoral transcriptional responses
Source: Front Immunol. 2023 Feb 10;14:1094326. doi: 10.3389/fimmu.2023.1094326 (PMC9950277; doi:10.3389/fimmu.2023.1094326)
Supplement: Supplementary Figure 1 — (A–C) Microscopic examination and immunostaining of hemolymph. Light microscopic examination of perfused hemolymph at (A) lower and (B) higher magnification showing multiple hemocyte populations. (C) Immunostaining of perfused hemocytes with WGA lectin (green), Vybrant CM-Dil (red), and Hoechst 33342 stains (blue). Scale bars as indicated. (D–I) Hemocyte populations in unfed ticks. Hemolymph was perfused from unfed male and female ticks and the (D) total and (E–I) differential hemocyte populations compared between unfed and partially fed male and female ticks using an improved Neaubeur chamber. Data were analyzed using unpaired t-tests in GraphPad Prism v8.4.1. *P < 0.05, **P < 0.01, ***P < 0.001, ****P < 0.0001. UF; unfed, PF; partially blood fed. [file Presentation_1.pdf]

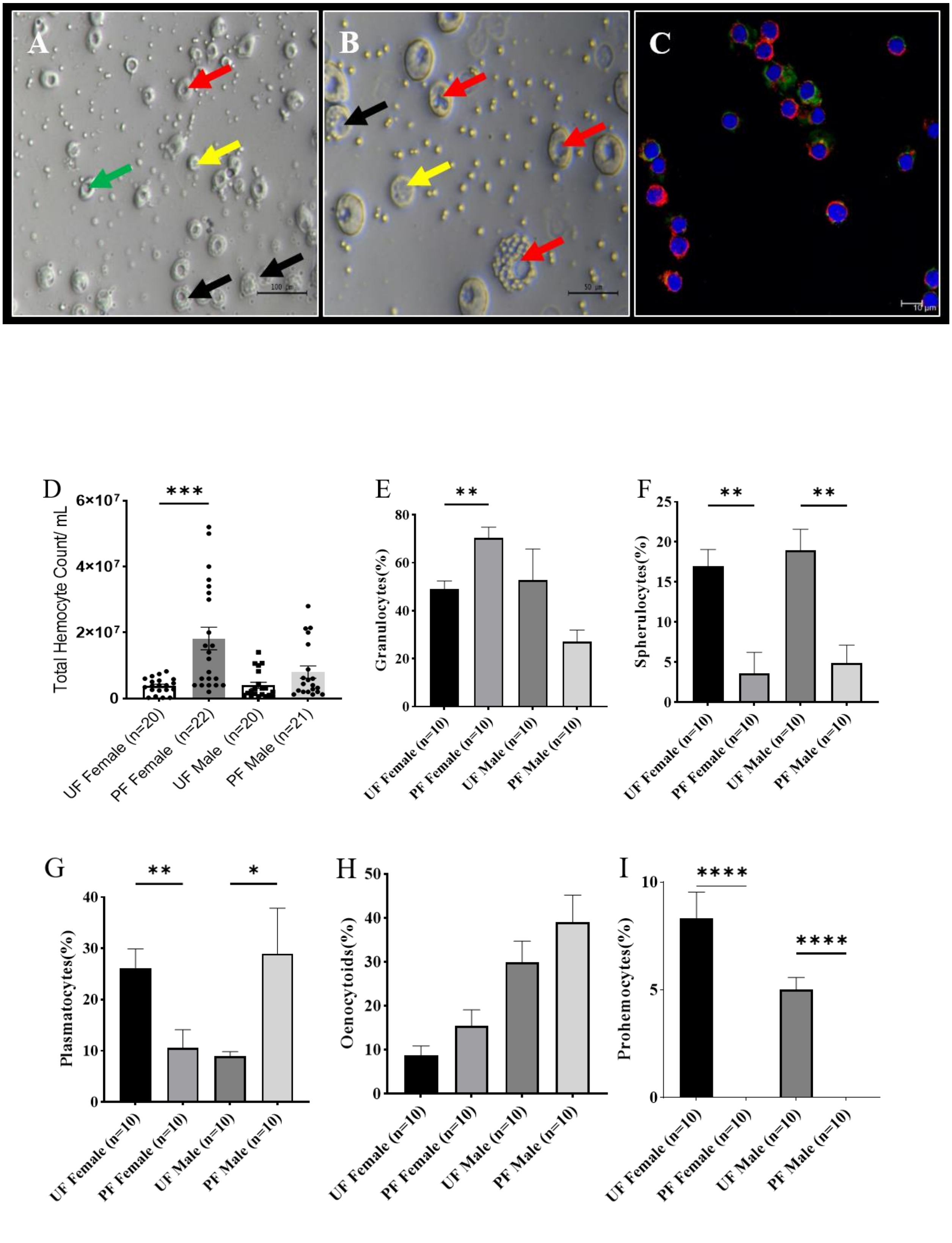

A
B
C
100 μm
50 μm
10 μm
D
***
Total Hemocyte Count/ mL
6×10^7
4×10^7
2×10^7
0
UF Female (n=20)
PF Female (n=22)
UF Male (n=20)
PF Male (n=21)
E
**
Granulocytes(%)
80
60
40
20
0
UF Female (n=10)
PF Female (n=10)
UF Male (n=10)
PF Male (n=10)
F
**
**
Spherulocytes(%)
25
20
15
10
5
0
UF Female (n=10)
PF Female (n=10)
UF Male (n=10)
PF Male (n=10)
G
**
*
Plasmatocytes(%)
40
30
20
10
0
UF Female (n=10)
PF Female (n=10)
UF Male (n=10)
PF Male (n=10)
H
Oenocytoids(%)
50
40
30
20
10
0
UF Female (n=10)
PF Female (n=10)
UF Male (n=10)
PF Male (n=10)
I
****
****
Prohemocytes(%)
10
5
0
UF Female (n=10)
PF Female (n=10)
UF Male (n=10)
PF Male (n=10)

Supplementary Figure S1A-C. Microscopic examination and immunostaining of hemolymph, and hemocyte populations within the tick. Light microscopic examination of perfused hemolymph at **(A)** lower and **(B)** higher magnification showing multiple hemocyte populations. **(C)** Immunostaining of perfused hemocytes with WGA lectin (green), Vybrant CM-Dil (red), and Hoechst 33342 stains (blue). Scale bars as indicated. Hemolymph was perfused from unfed male and female ticks and the **(D)** total and **(E-I)** differential hemocyte populations compared between unfed and partially fed male and female ticks using an improved Neaubeur chamber. Data were analyzed using unpaired t-tests in GraphPad Prism v8.4.1. *$P < 0.05$, **$P < 0.01$, ***$P < 0.001$, ****$P < 0.0001$. UF; unfed, PF; partially blood fed.

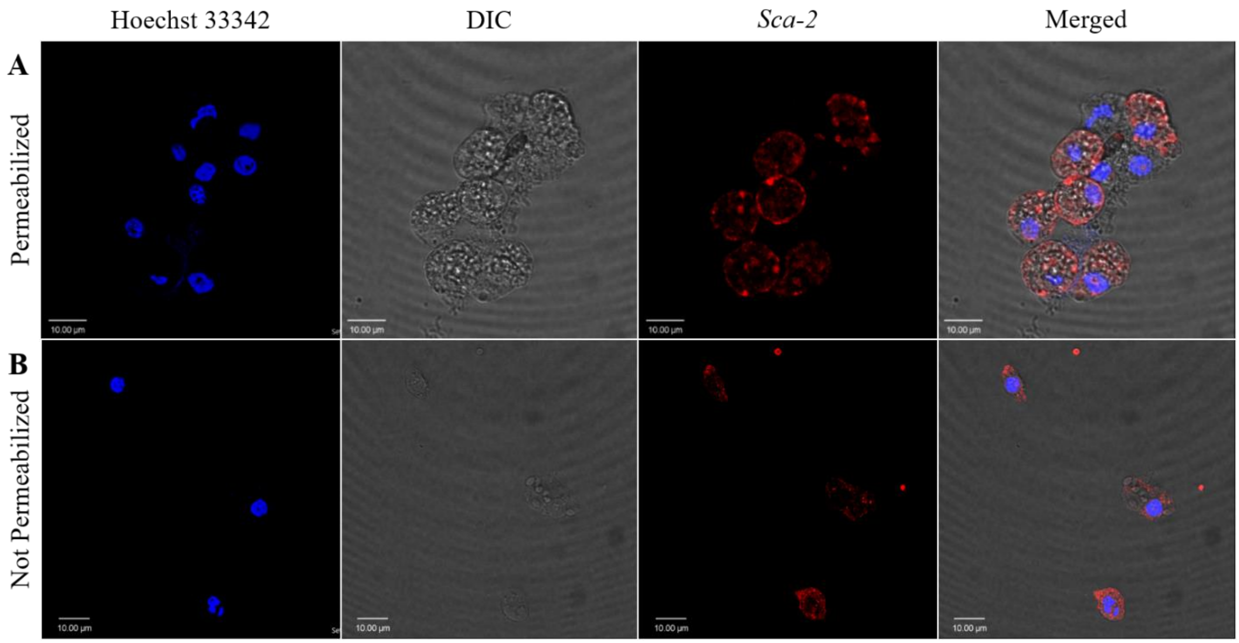

Supplementary Figure S2A and B. Confocal images of phagocytic hemocytes infected with *Rickettsia parkeri*. Representative confocal images of immunofluorescence staining for *R. parkeri Sca2* protein in hemolymph of *R. parkeri*-infected *Am. maculatum*. Hemocytes were either **(A)** permeabilized with Triton-X or **(B)** not permeabilized before incubation with *Sca2*-specific antibodies (red) and Hoechst 33342 (blue).

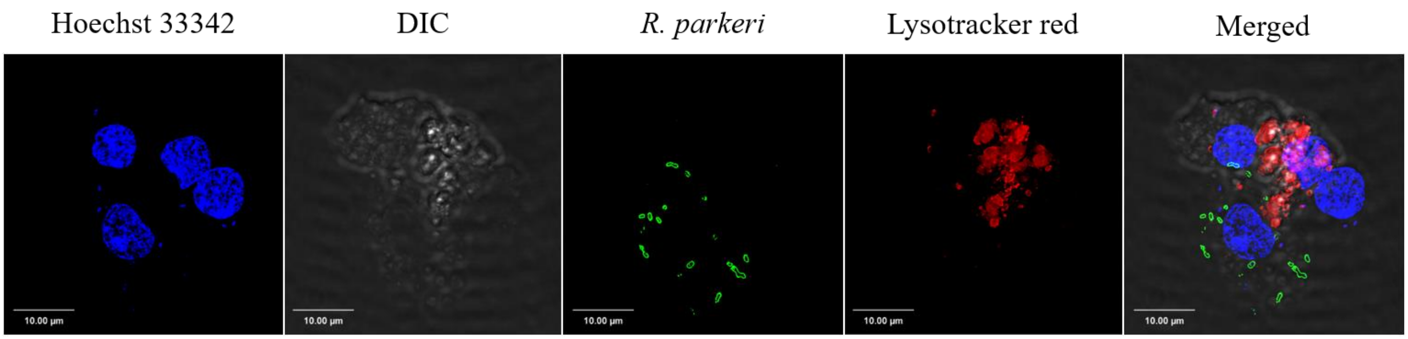

Supplementary Figure S3. LysoTraker Red labeling of lysosomal compartments in ticks fed with *R. parkeri*. Fluorescent microscopic images of hemocytes from *R. parkeri* infected ticks stained with LysoTracker Red (a lysosomal marker) 24 hours after infection. Hemocytes were incubated with primary antibodies against R. parkeri outer membrane protein (green) and Hoechst 33342 (blue). Scale bar = 10 µm.

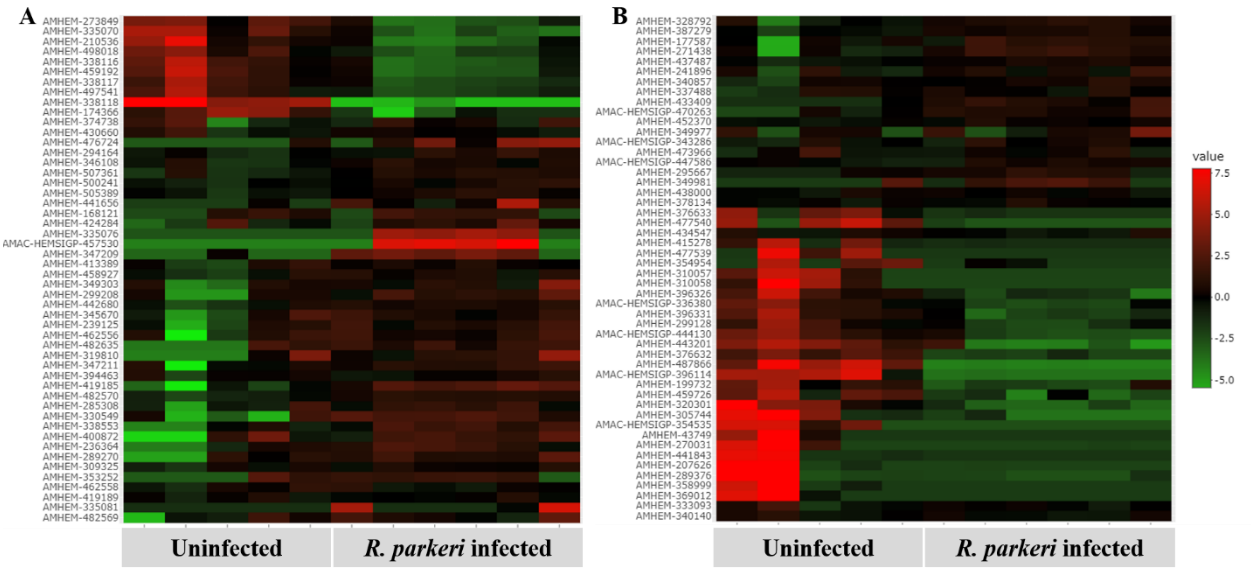

Supplementary Figure S4A and B. Heatmaps of RNA-seq expression data showing hematopoietic and cellular function genes differentially regulated in *R. parkeri*-infected compared with uninfected hemocytes. Several of these transcripts include **(A)** transcriptional and **(B)** humoral regulators of hemocyte differentiation and maturation. The scale bar shows the $\log_2$fold expression value of the transcripts.

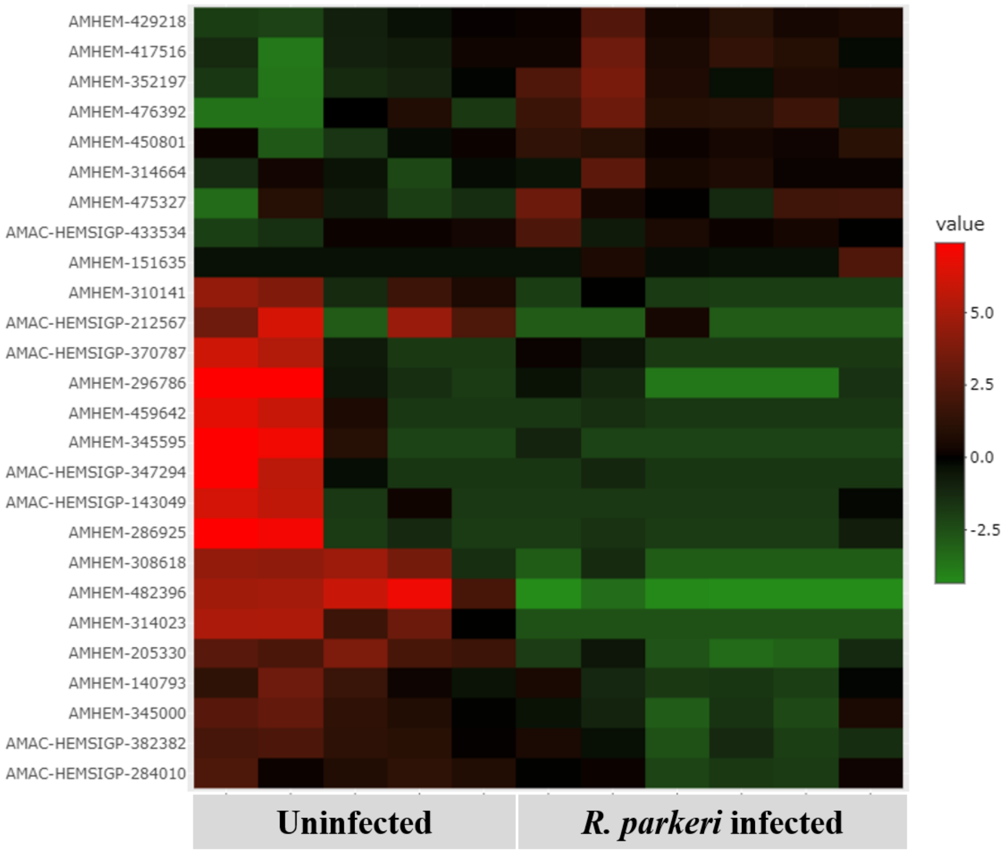

Supplementary Figure S5. Heatmap of RNA-seq expression data showing differentially expressed transcripts in the toll-signaling pathway in *R. parkeri* infected and uninfected hemocytes. Six of the nine Toll genes were identified including eight *PGRP* transcripts. Transcripts encoding *GNBP*, *tube*, and *pelle* were, however, not present. The scale bar to the right shows the $\log_2$fold expression value of the transcripts.

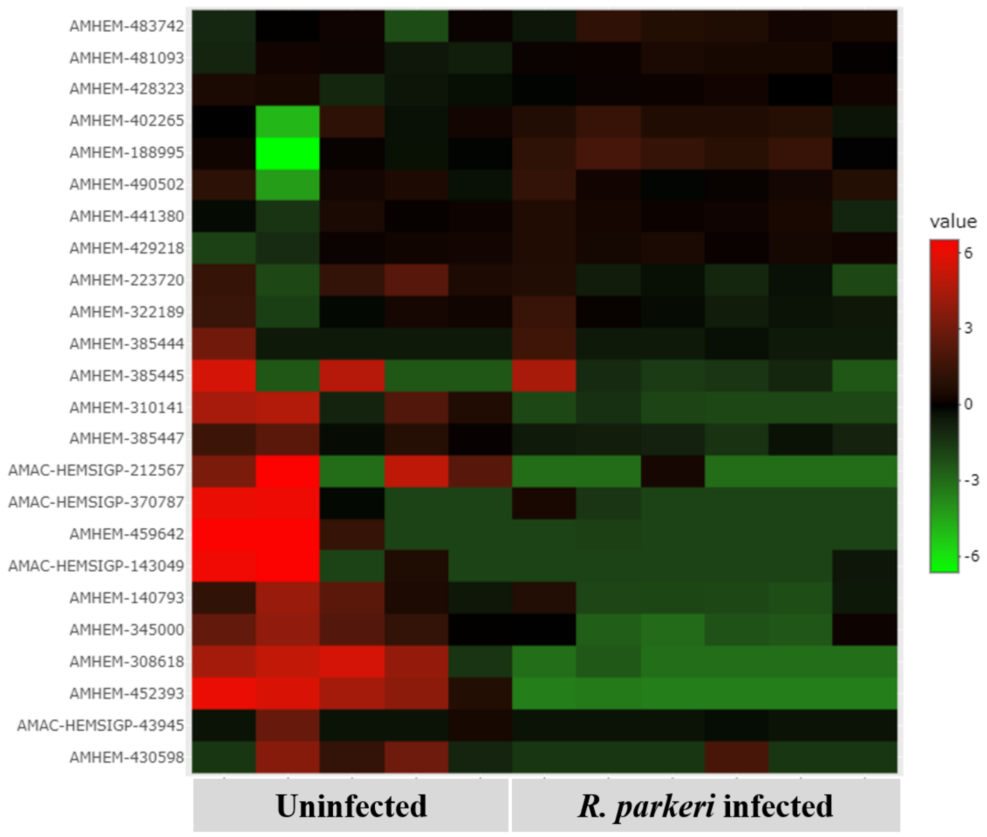

Supplementary Figure S6. Heatmap of RNA-seq expression data showing differentially expressed transcripts in the IMD signaling pathway in *R. parkeri*-infected and uninfected hemocytes. Sequences encoding *IMD*, Fas-associated via death domain (*FADD*). and death-related ced-3/Nedd2-like caspase (*DREDD*) genes were absent from our data. The scale bar to the right shows the log_2fold expression value of the transcripts.
